# Development and Validation of an *Haemophilus influenzae* Supragenome Hybridization (SGH) Array for Transcriptomic Analyses

**DOI:** 10.1371/journal.pone.0105493

**Published:** 2014-10-07

**Authors:** Benjamin A. Janto, N. Luisa Hiller, Rory A. Eutsey, Margaret E. Dahlgren, Joshua P. Earl, Evan Powell, Azad Ahmed, Fen Z. Hu, Garth D. Ehrlich

**Affiliations:** 1 Center for Genomic Sciences, Allegheny-Singer Research Institute, Pittsburgh, Pennsylvania, United States of America; 2 Department of Microbiology and Immunology, Drexel University College of Medicine, Allegheny Campus, Pittsburgh, Pennsylvania, United States of America; 3 Department of Biological Sciences, Carnegie Mellon University, Pittsburgh, Pennsylvania, United States of America; 4 Department of Otolaryngology Head and Neck Surgery, Drexel University College of Medicine, Allegheny Campus, Pittsburgh, Pennsylvania, United States of America; University of Bonn, Bonn-Aachen International Center for IT, Germany

## Abstract

We previously carried out the design and testing of a custom-built *Haemophilus influenzae* supragenome hybridization (SGH) array that contains probe sequences to 2,890 gene clusters identified by whole genome sequencing of 24 strains of *H. influenzae*. The array was originally designed as a tool to interrogate the gene content of large numbers of clinical isolates without the need for sequencing, however, the data obtained is quantitative and is thus suitable for transcriptomic analyses. In the current study RNA was extracted from *H. influenzae* strain CZ4126/02 (which was not included in the design of the array) converted to cDNA, and labelled and hybridized to the SGH arrays to assess the quality and reproducibility of data obtained from these custom-designed chips to serve as a tool for transcriptomics. Three types of experimental replicates were analyzed with all showing very high degrees of correlation, thus validating both the array and the methods used for RNA profiling. A custom filtering pipeline for two-condition unpaired data using five metrics was developed to minimize variability within replicates and to maximize the identification of the most significant true transcriptional differences between two samples. These methods can be extended to transcriptional analysis of other bacterial species utilizing supragenome-based arrays.

## Introduction

The tremendous advancement of sequencing technologies combined with rapid reductions in associated costs has allowed researchers not only to sequence more diverse organisms but also to sample individual bacterial species with much greater resolution. Extensive strain sequencing has led to the realization and appreciation that most bacterial species harbour enormous genomic diversity among strains [1]–[10] This diversity is manifested in small scale as single nucleotide polymorphisms (SNPs), and also the more dramatic swapping in and out of entire genes and/or operons through the process of horizontal gene transfer as predicted by the Distributed Genome Hypothesis [11]–[12]. Analyses of multiple genomes from individual bacterial species have led to the recognition that there exists a supragenome [11], [13] or pan-genome [1] at the species-level that is far larger than the genome of any single strain. The supragenome is composed of the core genome (those genes shared among all strains, and the distributed/accessory genome (those genes that are present in only a subset of strains). The ability to take up and incorporate DNA from the distributed genome by sampling from other strains’ DNA during polyclonal infections has been hypothesized to give these organisms an important mechanism for rapid diversity generation [11], [14]–[17]. This genetic diversity manifests as phenotypic diversity as different strains within the same species have been found to possess enormous differences in complex processes such as quorum sensing, biofilm formation and pathogenesis [12], [17]–[21] (Janto et al., Kress-Bennett et al. unpublished observations).

*Haemophilus influenzae* (Hi) is one such species of bacteria that has been demonstrated to possess enormous genomic variability [2], [5], [22]. These bacteria are commensals of the human respiratory tract but some have pathogenic potential. Un-encapsulated non-typeable Hi (NTHi) are most often associated with localized disease such as chronic obstructive pulmonary disease (COPD) [23]–[27], otorrhea [21], chronic otitis media with effusion (COME) and acute otitis media (AOM) [27]–[33], however, they are increasingly being found as the major source of invasive disease [34]–[38]. Individual NTHi strains share only ∼80% of their ∼1,800 genes with all other strains (core genes) with the rest being distributed (or accessory) genes [2]. The Finite Supragenome Model [2], [5] predicts the Hi supragenome to contain 4547 genes of which only ∼33% represent the core genome while the rest are present at various other frequencies among strains within the species [22]. The tremendous genic variability among NTHi strains presents a significant challenge when studying whole genome transcriptional patterns among many different strains. Traditionally, genic content must be known *a priori* in order to target genes with sequence-specific probes for measurement. Since two different strains might at a minimum share only the core genes, an array of probes designed for genes found in any single strain does not appropriately represent the species and therefore a significant amount of information will be lost (hundreds of genes) when using an array developed from a single strain. Thus, a more robust strategy for the design of bacterial microarrays is to use probes based on defined supragenomic sequences.

We previously designed and tested an *H. influenzae* supragenome hybridization (SGH) array in order to perform DNA-DNA hybridizations for the purpose of determining gene content in unsequenced strains [22]. This array was designed based on 3,100 genes that were identified in whole genome sequencing (WGS) of 24 geographically and clinically diverse NTHi strains and which includes >98% of all non-rare (ν>0.1) genes. Since genes are either present or absent from genomic DNA (gDNA) of any given strain, the signal obtained for each probe is essentially binary and a signal threshold cut-off was used to determine whether a gene was present or not. Nevertheless the data collected is quantitative and these arrays can also be used to hybridize labelled RNA instead of DNA thereby acting as a transcriptomic tool. Here we report the testing and validation of these custom SGH arrays for this application, as well as the design of an analysis pipeline for suggested use.

## Materials and Methods

### Design of the *H. influenzae* supragenome hybridization (SGH) array

Design and testing of the *H. influenzae* supragenome hybridization (SGH) array is described by [22]. Briefly, annotations from 24 sequenced *H. influenzae* strains were clustered using a custom supragenome pipeline to obtain unique clusters of genes [14]. NimbleGen probe design software was used to design between three and thirteen, 60 mer probe sequences to the longest sequence in each gene subcluster. Probes were tested and graded *in silico* based on uniqueness, distribution and probe manufacturing parameters. In all, 31,307 *H. influenzae* specific probes were synthesized by Roche/NimbleGen. Each array contained duplicates of each probe and each slide contained 12 arrays. An additional 185 negative control probes based on *Streptococcus pneumoniae* chromosomal sequences were also attached to the slides in duplicate and a further 9,053 random sequence probes were included. These arrays containing a total of 72,037 probes (72 K) are referred to as the SGH arrays.

### Genomic Hybridization

Genomic DNA (gDNA) was isolated from strain CZ4126/02 and Cy3-labeled using a NimbleGen One-Color DNA Labeling Kit. NimbleGen Hybridization Kits and Sample Tracking Control Kits were used to hybridize this labeled DNA to the custom-designed *H. influenzae* SGH arrays as well as for array washing. Images were acquired on an Axon Instruments GenePix 4200AL array scanner.

### Genomic Hybridization data processing

Images were processed and data were normalized within chips using a Robust Multichip Average (RMA) algorithm and quantile normalization via the NimbleScan software v2.5 [30], [40]. Raw data was converted into gene possession or absence by applying a combination of an expression threshold (1.5X the median background value in log_2_ scale) and a measure of probe variance [22]. Subclusters producing a signal above this value were set to a value of 1 (present) and subclusters with values below this value were set to a value of 0 (absent). The list of present subclusters was then used as a reference list for filtering transcription-based microarray data.

### Experimental design and sample collection for two-condition transcriptional microarray analyses

Parallel work has focused on the role of AI-2 signalling in Hi by comparative studies between CZ4126/02 and an AI-2 sensing mutant (CZ4126/02ΔLsr::Cm^r^ [KO]) (Janto et al. unpublished observations). These strains were used to test and validate the use of the SGH Array for unpaired two-condition transcriptional microarray analysis. The CZ4126/02 WT and its cognate KO strain were grown in two different media (BHI and CDM) and sampled at multiple time points (the combinations of which are referred to here as “conditions”, **Table S3**) for RNA extraction. The RNAs were converted to cDNA, Cy-3 labelled and hybridized to the *H. influenzae* SGH arrays as described below. For each condition, RNA samples were collected twice which are referred to as replicates A and B. Each condition/replicate was hybridized on two different SGH arrays referred to as chip 1 and chip 2. Finally each array outputs separate information for two duplicate probe-sets referred to as probe-set 1 and probe-set 2. All transcriptional data in this study has been deposited in NCBI's Gene Expression Omnibus (GEO) [41] and are accessible through GEO Series accession number GSE41690 [42].

### *H. influenzae* culture media

Brain-Heart Infusion broth (BHI - Oxoid) was made using 37 g of powdered media/L and supplemented with hemin (Sigma-Aldrich) to a final concentration of 10 µg/mL and β-nicotinamide adenine dinucleotide (β-NAD) to a final concentration of 2 µg/mL. Chemically Defined Media (CDM) was made exactly as described [43] with the following minor catalog change (Dr. Arnold Smith, personal communication): 1X RPMI 1640 with glutamine and 25 mM HEPES (Gibco, catalog #22400-089).

### Bacterial growth for RNA extraction

For microarray experiments frozen stocks were used to inoculate BHI plates that were incubated overnight at 37°C with 5% CO_2_. Isolated colonies from these plates were used to inoculate 5 mL BHI cultures that were incubated overnight at 37°C with shaking at 200 rpm. The cultures were diluted to an OD_A600_ of 0.02 in 40 mL BHI or CDM. At selected time-points (**Table S3**), 1 mL culture samples were collected and transferred immediately into 2 mL RNAProtect (Qiagen). Samples were incubated for 10 minutes at room temperature and then stored overnight at 4°C.

### RNA extraction and quality check

Samples stored in RNAProtect were spun for 10 minutes at 2,500×g (Sorvall RT-7), the supernatant removed and the cell pellets resuspended in 100 µL of 1X Tris-EDTA (TE) +1 mg/mL lysozyme (Worthington Biochemical) and 1 mg/mL proteinase K (Qiagen). RNA was extracted using a Qiagen RNeasy Mini Plus kit with the standard protocol including genomic DNA (gDNA) eliminator columns. The eluted RNA (∼85 µL) was DNased by adding 10 µL 10X TurboDNase buffer and 5 µL TurboDNase (2 units/µL) (Ambion) and incubating at 37°C for 1.5 hours. 2 µL more TurboDNase was added and incubation continued for an additional 1.5 hours. The DNased RNA samples were cleaned by passing the samples through the RNeasy protocol a second time (including the gDNA eliminator column steps). Samples were eluted in nuclease free (n.f.) water, quantitated on a Nanodrop 1000 spectrophotometer and stored at −80°C. 200 ng of each RNA sample was run on an Agilent 2100 Bioanalyzer using RNA Nano6000 chips to check for RNA degradation. We performed paired reverse transcription reactions on every RNA sample where one reaction received reverse transcriptase (+RT, M-MLV, Promega) and the other did not (−RT). Both reactions were PCR amplified using primers directed against a housekeeping gene (GAPDH) and observation of amplification in the +RT reaction as well as lack of amplification in the −RT reaction verified removal of gDNA from each RNA sample.

### qRT-PCR

Single-stranded cDNA was synthesized from the extracted RNA samples using the Roche Transcriptor First Strand Synthesis kit. Specific primers for the housekeeping and experimental genes were designed using Roche Probe Finder online software in order to design ∼75 bp amplicons. qRT-PCR was performed on the Roche Light Cycler 480 using a SYBR green master mix. Reactions were performed in a 20 µl volume containing 2 µl cDNA (1∶5 dilution) and primers at 0.5 µm each. Primer efficiency was determined by testing all primer pairs ahead of time with gDNA template. All reactions were measured in triplicate. The experimental data were normalized using the hpr and ldhA genes as internal standards. Independent data analysis was carried out using both the Pfaffl-ΔΔC_T_ method with the Roche Light Cycler software as well as a linear regression method using the LinRegPCR [44] software package. Fold changes presented are the mean results from both methods of analysis and from normalization against both of the housekeeping genes.

### Generation of labelled double-stranded cDNA for SGH array hybridization

First and second-strand cDNA synthesis was performed using a SuperScript One-Cycle cDNA Kit (Invitrogen) as outlined in the NimbleGen Microarray Experienced User’s Guide including RNaseA and cDNA precipitation steps. 1 µg of cDNA was Cy3-labeled using a NimbleGen One-Color DNA Labeling Kit. NimbleGen Hybridization Kits and Sample Tracking Control Kits were used to hybridize the labelled cDNA to the custom-designed *H. influenzae* SGH arrays as well as for array washing. Images were acquired on an Axon Instruments GenePix 4200AL array scanner.

### Analysis of microarrays

Images were processed to.pair files containing expression values for both sets of duplicate probes representing all the subclusters on the *H. influenzae* SGH array using the NimbleScan software. These.pair tables were merged with a reference list of subclusters that had been determined to be present in the CZ4126/02 genome (see above) in order to remove non-relevant probe/subcluster data. These parsed.pair files were then normalized within and across chips using a Robust Multichip Average (RMA) algorithm and quantile normalization using the NimbleScan v2.5 software followed by a median polish whereby the 3 to 13 probe values/subcluster were condensed to a single value (in duplicate) [39], [40]. Duplicate probe-set values were treated as independent replicates. For comparison of technical and biological replicate data, CyberT was used to obtain Bayesian corrected p-values, Bonferroni corrected p-values and Benjamini-Hochberg values [45]. Significance Analysis of Microarrays (SAM v3.0) was used to obtain lists of genes with associated permutation-based false discovery rates (FDR) [46]. These data were combined and filtered in the following order: 1) SAM FDR <10%, Bayesian p-values<.05, Benjamini-Hochberg FDR<10%, Bonferroni corrected p-value<.05, raw values in at least one of the two conditions being compared >256 normalized intensity.

## Results

### Removal of non-relevant subclusters for microarray analysis

The custom-designed *H. influenzae* SGH array contains 31,307 unique probes that target 2,890 of the 3,100 gene clusters identified in 24 geographically diverse clinical strains. Gene clusters were further subdivided into “subclusters” with more stringent alignment parameters in order to capture allelic differences within more variable genes. The power of this array is its ability to capture information for any strain of *H. influenzae* since the probes represent the majority of the predicted supragenome (>85% of all “non-rare” genes. Non-rare genes are defined as those that are present in more than 10% of Hi strains) [22]. However, since a large proportion of the gene probes present in the SGH array do not correspond with any gene for any given single strain, once a strain is selected for study, an *in silico* analysis should be performed to ensure that only the relevant subset of probes is included in the final analysis. This is important for the purposes of obtaining a Gaussian distribution of data needed for both normalization and statistical testing. Therefore, it is necessary to remove all data from so-called “non-relevant” gene clusters, defined as those present in one of the 24 strains used to design the array but not present in the strain being interrogated.

For testing purposes we used a strain (CZ4126/02) that was not included in the design of the *H. influenzae* SGH array. Our first task was to determine the gene content of this strain for the purposes of removing non-relevant data later. This we accomplished by two methods 1) WGS of CZ4126/02 and mapping of identified genes back to the SGH array clusters (Janto et al. unpublished observations) and 2) hybridization of genomic DNA (gDNA) to the SGH array and application of a signal threshold to determine whether a gene was present or not [22]. A comparison of the WGS and SGH data sets from strain CZ4126/02 revealed that 2805/2890 (97%) of the identified gene clusters were in agreement between the two methods. Using WGS as the gold standard we identified 39 false positives (some of which could potentially be true positives present in WGS contig gaps) and 46 false negatives. In addition, we found only four genes in the WGS that were not represented on the SGH array [22]. Because of this accuracy we used SGH data for the purposes of removing data not relevant to strain CZ4126/02.

**Figure 1:**
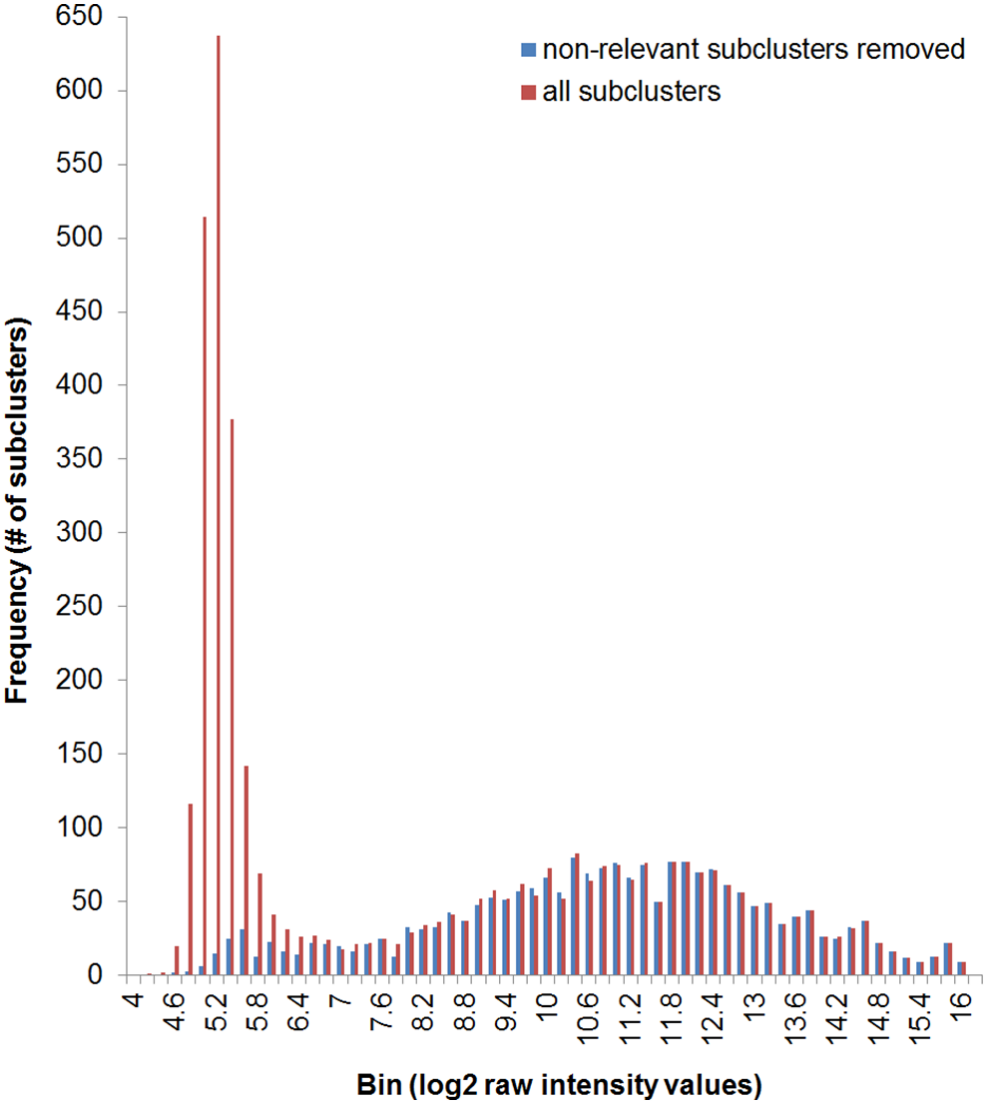
Data distribution with and without removal of non-relevant subclusters. Data from condition 3 (WT CZ4126/02 grown in in CDM media to OD_A600_ 1.0), replicate A, chip 1. Intensity values are binned by 0.2 in log_2_ scale. Non-relevant subclusters are defined as genes that are not present in the strain being interrogated and thus are uninformative (and detrimental) to the analysis.

From the SGH gene possession experiment, hybridized CZ4126/02 gDNA gave a positive signal for 1702 of the 2890 total gene clusters and 2194 of the 4052 total gene subclusters represented on the array. This list of 2194 gene subclusters was then merged with the raw output from transcriptional experiments to isolate data only associated with those CZ4126/02 strain-specific subclusters. All transcriptional data in this study has been deposited in NCBI's Gene Expression Omnibus (GEO) [41] and are accessible through GEO Series accession number GSE41690 [42]. A representative histogram of the distribution of log_2_ transformed raw intensity values obtained before and after removal of non-relevant subclusters is shown in **Figure 1**. A summary of the distributions of each data set (relevant and non-relevant) compared to random and control data at the probe-level is presented in **Table 1**.

**Table 1:** Comparison of the distributions of raw data and filtered sub sets of probe-level data.

	mean	median	max	min	N
ALL probe-set 1	6.85	5.49	15.99	3.45	31,307
ALL probe-set 2	6.85	5.49	15.99	3.43	31,307
CZ specific probe-set 1	10.22	10.42	15.99	3.79	10,161
CZ specific probe-set 2	10.23	10.44	15.99	3.86	10,161
Not CZ probe-set 1	5.31	5.16	14.62	3.69	21,146
Not CZ probe-set 2	5.31	5.16	14.65	3.68	21,146
Negative probe-set 1	5.15	5.09	6.65	3.71	185
Negative probe-set 2	5.27	5.17	7.46	3.78	185
Random control probes	5.12	5.01	10.83	3.53	9,053

Expression data in log_2_ from condition 3, replicate A, chip 1. Raw output (ALL) was filtered using SGH data to produce subsets of data containing subclusters (and associated probes) present in CZ4126/02 (CZ specific) and subclusters not present in CZ4126/02 (Not CZ). Normalization was performed after filtering. Negative: probes synthesized from *Streptococcus pneumoniae* genes expected to be absent in *H. influenzae*. N: number of probes in each set. Probes are synthesized in duplicate on each chip (probe-set 1, probe-set 2).

After removal of non-relevant subcluster data, the remaining data was normalized between and across chips where necessary using the NimbleGen NimbleScan software. In the same representative sample we found only fourteen false positives (14/2194 or 0.64%) (gene subclusters that gave a transcriptional signal significantly above background that did not give a SGH signal over threshold) illustrating the consistency across methods of analysis.

### Step-wise filtering with statistical tests and testing on technical and biological replicates

**Figure 2:**
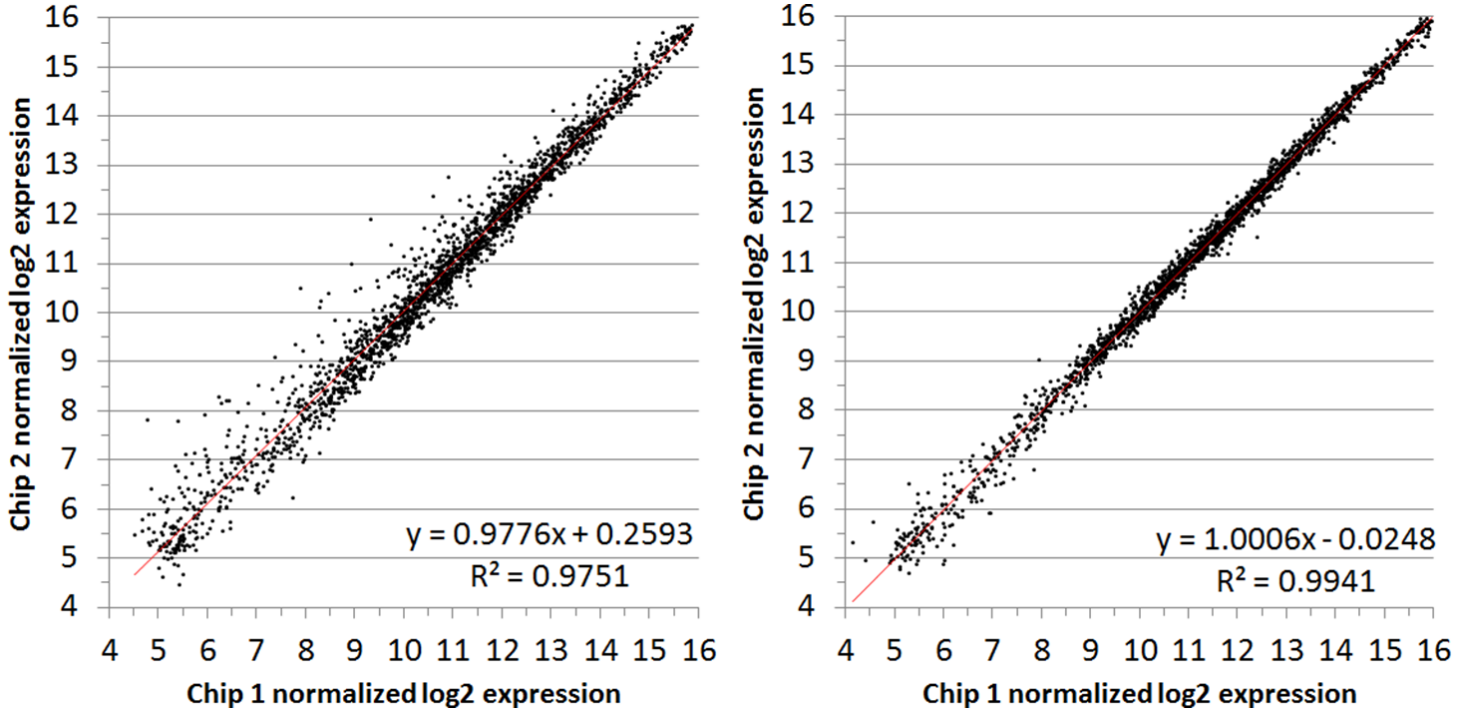
Comparison of chip replicate values for RNA from condition 4. Log_2_ expression values (average of two probe-sets) from the same RNA samples run on two different chips: chip1 (x-axes) and chip2 (y-axes). Condition 4 (CZ4126/02ΔLsr::Cm^r^ grown in CDM media to OD_A600_ 1.0), replicate A (left) and replicate B (right).

In analyzing the data here, a Bayesian-corrected variance was applied and t-tests were performed using the web-based microarray analysis tool, CyberT [45]. We found that using a Bayesian p-value cut-off of 0.05 alone is not stringent enough as it results in reporting of a large number of false positives. This is illustrated by comparing the technical replicates for condition 4 (the same RNA sample run on two different chips), in this case biological replicate B, chip 1 vs. chip 2, which produced nearly identical results, with a R^2^ of.9941 (**Figure 2**). Submitting this comparison to CyberT and obtaining Bayesian corrected p-values results in a list of 120 subclusters with p<0.05, each of them a false positive (**Table S2**). In this same dataset applying a fold change cut-off alone is similarly inappropriate. In this comparison of a technical replicate, 30 subclusters are found with a fold change >1.5, all false positives (**Table S3**). Twenty-three (23)/30 of these subclusters have expression values below 2^8^ (256) raw intensity on both chips. These two parameters applied together (fold changes and t-tests) give some measure of biological and statistical significance that compensate for each others’ weaknesses as far fewer clusters meet both cut-offs of fold >1.5 and p<0.05 than either alone, however, there are still several that slip through as false positives.

Therefore, a permutation-based false discovery rate (FDR) was calculated with the Significance Analysis of Microarrays (SAM) excel plug-in [46]. An FDR (q-value) of 10% calculated by SAM was used for this filtering step. A non-permutation-based estimate of the FDR was also used as an additional filter again with a cut-off of 10% (0.1) (Benjamini-Hochberg [BH]). The extremely stringent Bonferroni-corrected p-value was used to identify only the most significant findings. In the technical replicate comparison discussed above (Condition 4, replicate B, chip 1 vs. chip 2), the application of any one of these three additional statistical filters (SAM FDR, BH FDR, or Bonferroni-corrected p-value) results in no significant findings between the technical replicates, which is the reality (**Table S1**).

Thus, a custom step-wise filtering process was developed and implemented roughly in order of stringency. This involved obtaining the set of genes associated with the SAM permutation-based FDR<10% and then removing genes with a Bayesian-corrected p-value>.05. In some samples the SAM FDR filter was more stringent than the Bayesian-corrected p-value, therefore, the order of filtering was flipped in these cases. After this, genes were removed that had a Benjamini-Hochberg FDR>10%. The remaining genes with Bonferroni corrected p-values<.05 were selected for the next step. Any findings with raw intensity values below 2^8^ (256 = ∼1.5X background in log_2_) in both conditions were not considered sufficiently above background levels and also removed. This step-wise application of filters complemented each test’s weaknesses and allowed us to observe lists of differentially regulated genes at varying levels of stringency. Fold change was not considered until after the final filter and is presented as is with no cut-off. In most cases this step-wise filtering resulted in final lists of genes up or downregulated by more than 1.5 fold which we consider to be reasonably biologically relevant.

### Technical replication for microarray studies

**Figure 3:**
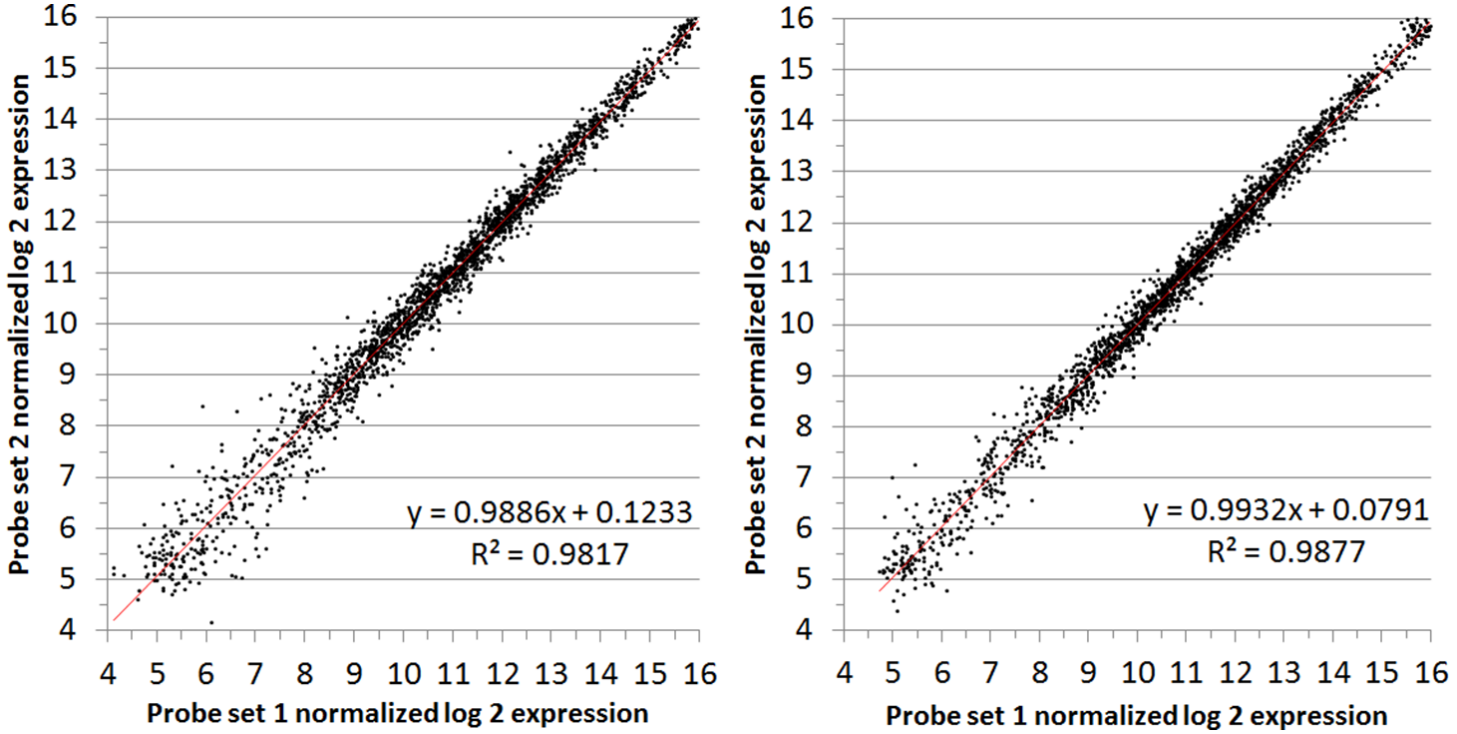
Comparison of probe replicate values within RNA from condition 3, replicate A. Log_2_ expression values from the two probe sets in condition 3 (WT CZ4126/02 grown in in CDM media to OD_A600_ 1.0), replicate A on two separate chips (left/right).

**Figure 4:**
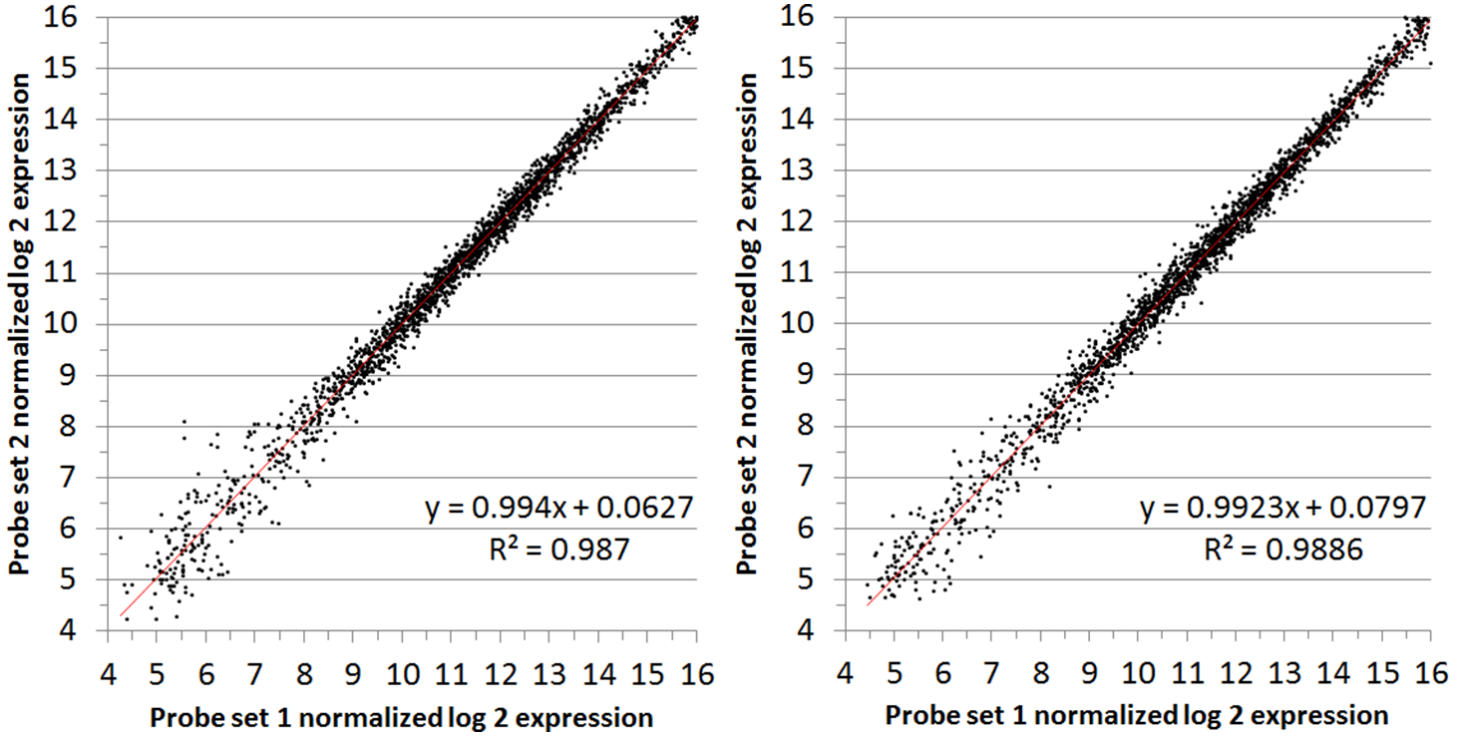
Comparison of probe replicate values within RNA from condition 3, replicate B. Log_2_ expression values from the two probe sets in condition 3 (WT CZ4126/02 grown in in CDM media to OD_A600_ 1.0), replicate B on two separate chips (left/right).

An exhaustive analysis of technical replicates was performed to assess the reproducibility of the *H. influenzae* SGH chips for transcriptomic analyses. Two levels of technical replication were applied. The first level is contained within the array design wherein each probe is represented in duplicate on each chip. Since each subcluster is represented by between 3 and 13 probes on each chip, two sets of 3–13 values are obtained per subcluster. The NimbleScan normalization process includes a median polish whereby the 3–13 probe values are condensed to a single value, which again is calculated in duplicate. We describe this type of technical replication as probe-replication. Therefore the first validation test was to evaluate these duplicate normalized probe values as shown in **Figures 3**, and **4** for two separate RNA samples from a single condition. Each figure displays two plots which represent the same RNA sample run on two separate chips. There is no major skew in any of the data as evidenced by the best-fit lines associated with very high correlation coefficients (R^2^) >0.98. Most variation occurs at expression values at or below background levels (log_2_ value of ∼5.5). Comparison of probe-sets was performed for an additional 9 conditions (18 RNA samples; 36 chips) the results of which are displayed in figures **S1–S18**.

**Figure 5:**
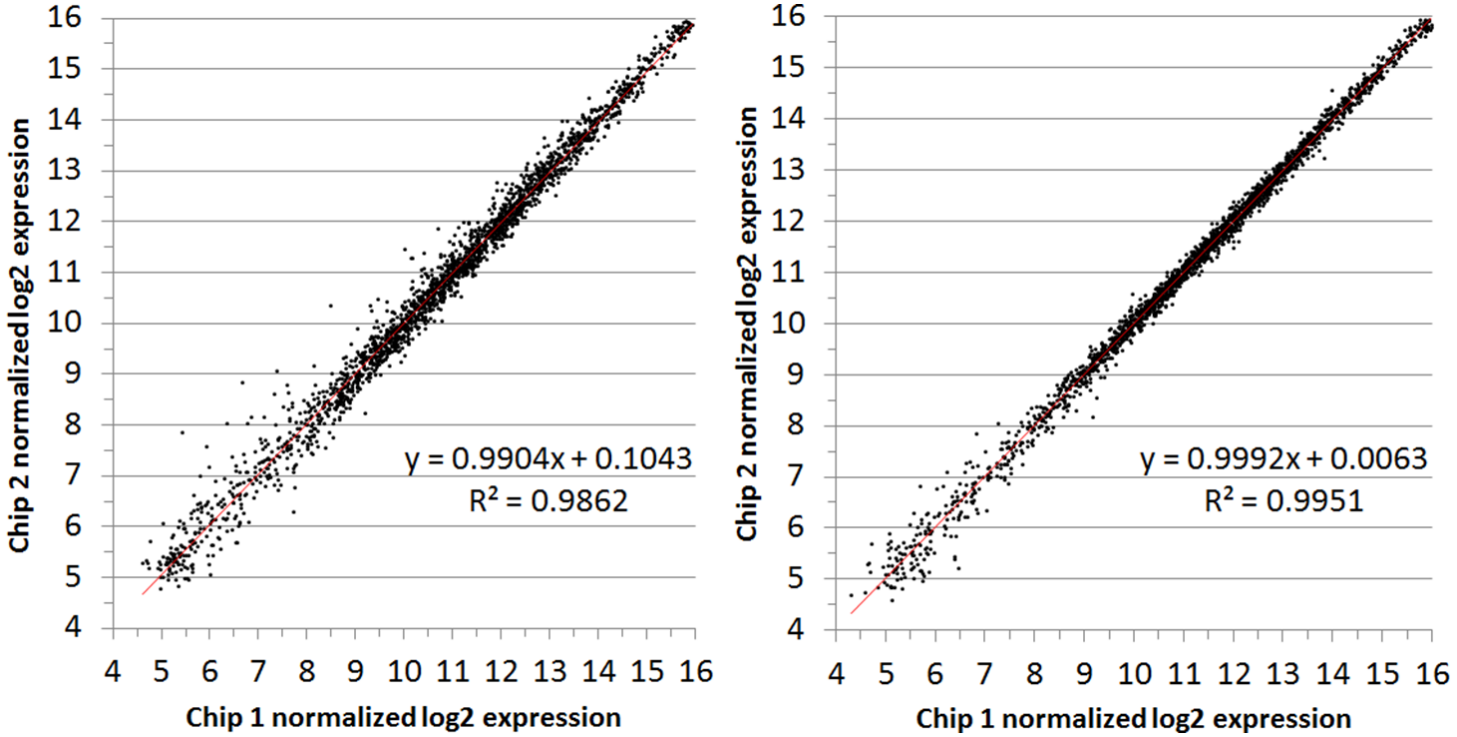
Comparison of chip replicate values for RNA from condition 3. Log_2_ expression values (average of the two probe-sets) from the same RNA samples run on two different chips: chip1 (x-axes) and chip2 (y-axes). Condition 3 (WT CZ4126/02 grown in in CDM media to OD_A600_ 1.0), replicate A (left) and replicate B (right).

A second level of technical replication was performed in microarray studies by hybridizing each RNA sample to two separate chips. Therefore this level of replication evaluated the reproducibility of the labeling, hybridization, scanning and normalization processes. Similar expression plots were generated by averaging the two duplicate-probe intensity values for chip 1 and plotting against the average for chip 2 (**Figures 1** and **5**). We observe that the inter-chip variability is just as low or lower as the probe variability with R^2^ values ranging from 0.9751 to as high as 0.9951. These extremely high correlation coefficients were found consistently throughout all of the transcriptomic studies performed and are presented in full in figures **S19–S26**.

### Biological Replication

**Figure 6:**
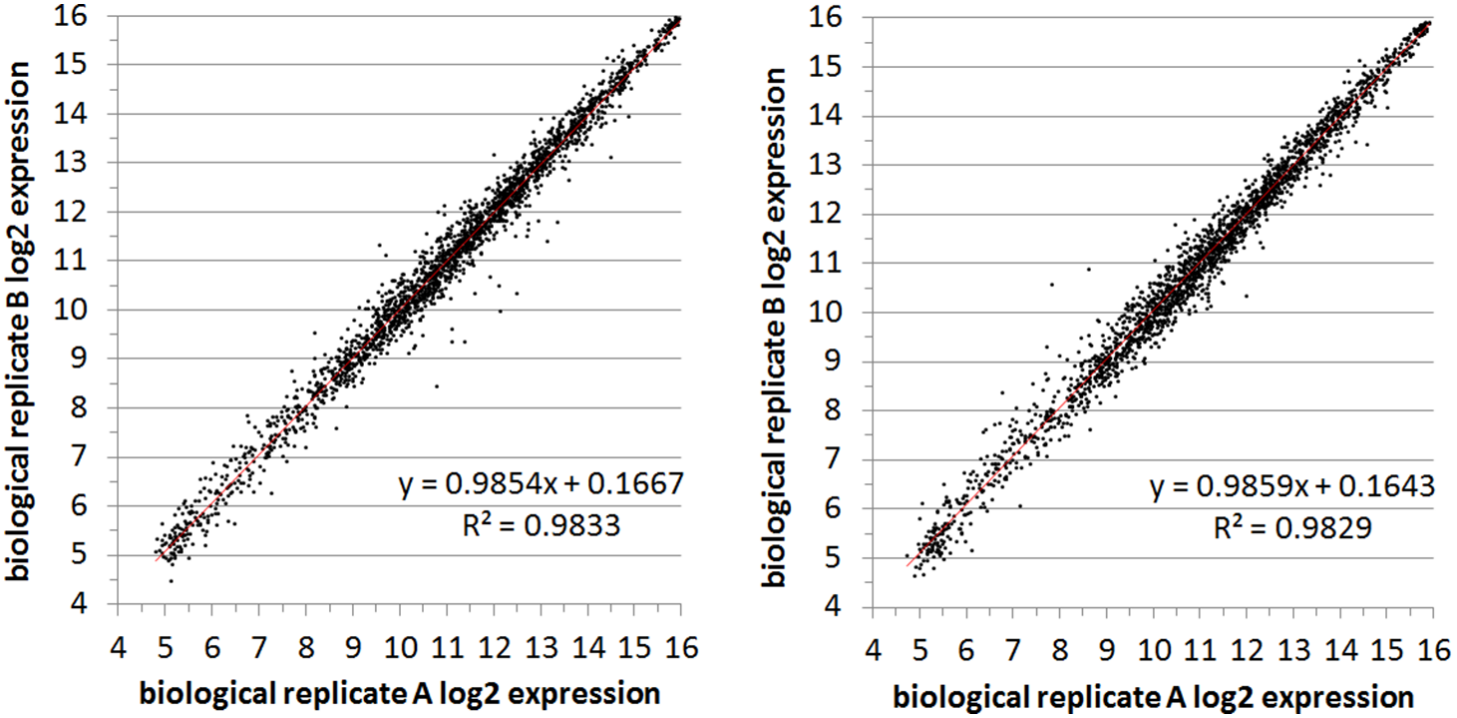
Representative biological replicate comparisons. Log_2_ expression values (averages of both probe-sets from both chips) from the two biological replicate samples for both condition 3 (left, WT CZ4126/02 grown in in CDM media to OD_A600_ 1.0) and condition 4 (right, CZ4126/02ΔLsr::Cm^r^ grown in CDM media to OD_A600_ 1.0).

**Figure 7:**
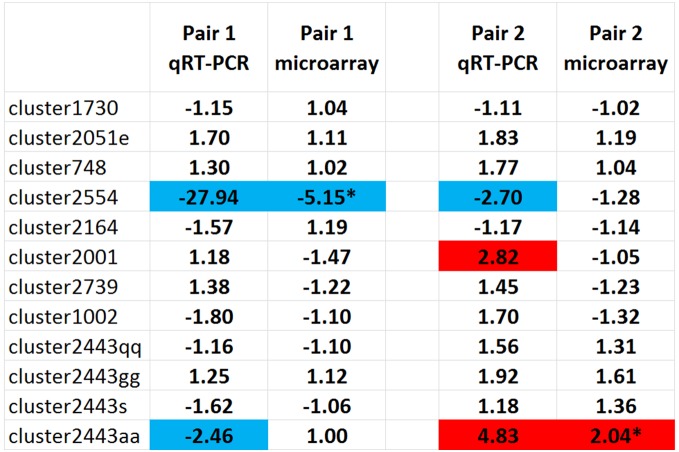
Comparison of SGH microarray analysis vs qRT-PCR in two biological replicate pairs. All values represent fold changes between biological replicates. Pair 1 = Condition 3 (WT CZ4126/02 grown in in CDM media to OD_A600_ 1.0) replicate A vs. replicate B. Pair 2 = Condition 4 (CZ4126/02ΔLsr::Cm^r^ grown in CDM media to OD_A600_ 1.0) replicate A vs. replicate B. qRT-PCR data are the average from normalization against two different house-keeping genes and two different methods of analysis (Pfaffl-ΔΔC_T_ and linear regression) *a statistically significant difference from SGH microarray analysis (passed all five filtering steps). Colors highlight fold changes above 2 (red) and below −2 (blue).

To establish whether the results are reproducible across different RNA samples, two separate cultures of CZ4126/02 were grown to obtain duplicate RNA samples under the same conditions (**Table S3**). In chemically defined media (CDM), R^2^-values ranged between 0.9751 and 0.9945 (**Figure 6**, Figures **S27 and S28**). When grown in the complex media, brain-heart infusion (BHI), the R^2^-values ranged between.9385 and.9696 (Figures **S29** and **S30**). Furthermore, we compared the results of our microarray analysis pipeline with qRT-PCR analysis on pairs of biological replicates (**Figure 7**). As these were biological replicates we did not expect to see any differences. However, our microarray analysis pipeline indicated that two genes (cluster2554 and cluster2443aa) were significantly differentially regulated between these particular biological replicates and this was confirmed in the qRT-PCR analysis. This demonstrates that comparable results are obtained between the two methods of analysis.

## Discussion

In this paper we repurpose an established CGH array for microarray-based transcriptional analysis of virtually any *H. influenzae* strain without the need for sequencing of the genome. The current format of the *H. influenzae* SGH array allows for 12 independent hybridizations (samples) per chip and here we describe a single-color fluorescence analysis pipeline. Strain-specific genome variability in terms of gene possession is captured by the SGH array [22] via-hybridization with genomic DNA. This provides all the information required for removal of non-relevant gene clusters during subsequent transcriptional analysis. While WGS provides more information than SGH it is not necessary for the purposes of transcriptional experiments. SGH produces excellent results more rapidly, at a lower cost and is more easily integrated because the reagents, the array itself and the data output are exactly the same in SGH and transcriptional experiments. For transcriptional experiments we estimate the costs per sample to be approximately $110 with a workflow of four days from sample collection to data output (including analyses). Compared with RNAseq this is a cheaper alternative at this time which can run several hundred dollars more for a small bacterial genome. Organisms with larger genomes require more sequencing and thus the cost per sample increases. Significantly adding to the cost and workflow of RNAseq is the requirement for pre-processing of RNA to remove ribosomal RNA before being sequenced. However, besides cost there are other considerations to be taken into account if deciding between these technologies. Although microarrays remain the cheaper option RNAseq has been demonstrated to have higher sensitivity and a higher dynamic range than microarray analysis [47]. If the objective is to detect genes expressed at very low levels RNAseq may be a better choice. Furthermore, because RNAseq is non-probe-based it allows measurement of all expressed transcripts without *a priori* knowledge of the genome content thereby bypassing any artifacts of gene annotation and potentially identifying novel gene transcripts. In contrast, the described SGH microarray is targeted and those targets are fixed, thus a small amount of data will inevitably be missing for a few genes in any given strain. However, we have demonstrated here that we are able to capture the vast majority of annotated genes in any *H. influenzae* strain by incorporating the supragenome into the design of the array [22]. Another major consideration is that microarray analysis methods and software are well-established and user-friendly while RNAseq data analysis has not reached the same level of development, requiring knowledge and expertise in handling large datasets as well as the command line.

Because of the multiple strain design of the SGH array some extra steps in data analysis are required that are not normally encountered in single organism microarrays. Primarily this involves removing hybridization data that is not relevant to the strain under investigation (ie probes that are designed to genes that are not present in that particular strain). A comparison of data distributions before and after the removal of non-relevant gene clusters confirmed that the transcriptional datasets are converted from non-Gaussian to Gaussian distributions (**Figure 1** **,** **Table 1**).

For sample comparison we developed an analysis pipeline to be used with the normalized strain-relevant data that incorporates two freely available microarray analysis tools (SAM and CyberT) that together incorporate four diverse methods of statistical analysis. The most logical measure of difference used to analyze transcriptional data is the fold change, obtained simply by dividing the sample means. The weakness in drawing conclusions using fold changes alone lies in the blindness to expression levels illustrated in **Figure S3**. To compensate, a statistical comparison of mean values using T-tests is commonly used. However, this test is influenced greatly by the variance of the samples, something that is determined by both the biology of the gene and also by the equipment and methods used to measure the sample. The Bayesian-corrected variance is a method for reducing noise in microarrays which can overwhelm biological differences with limited sampling. This method infers single gene variance based on the variance of other similarly expressed genes. We found in developing our transcriptional comparison pipeline that relying on calculated Bayesian-corrected p-values in conjunction with fold change cut-offs was not stringent enough as false positives were still being detected between replicate samples (**Tables S2, S3**).

To further control for the discovery of false positives (FDR) we used both permutation and non-permutation based methods using SAM and CyberT. One weakness of SAM is that it is inappropriate for small numbers of replicates. This is due to the fact that the modified p-values (q-values) are calculated based on the number of times the software can randomly rearrange the replicate values (permutations). Thus SAM’s power, accuracy and significance increase with increased replication.

As a final filtering step we used Bonferroni-corrected p-values calculated by CyberT. In many microarray analyses applying the Bonferroni correction is not performed because it is too stringent and often removes data that is known to differ between two samples (false negatives). In these *H. influenzae* microarray studies, however, we found that it was appropriate and useful for removing many genes that had fold changes under 1.5 but were still found significant by the other tests as well as for removing genes for which there was high variance between biological samples. These four filtering steps (T-tests, permutation-based FDR, BH FDR and Bonferroni-corrected T-tests) used together in conjunction with a raw hybridization value cut-off provide a robust, flexible method for two-condition microarray analyses.

Replicate samples were examined in detail and conclusively demonstrated that duplicate probe sets provide consistent repeatable quantitative data (**Figure 3** **–** **4** **, S1–S18**). We also found that technical replication by hybridization of the same cDNA samples on two different chips was just as robust as probe replication (**Figures 1** **,** **5** **, S19–S26**). The correlation between biological replicate experiments is also extremely high, especially when bacteria were grown in the defined media, CDM, where the growth conditions are more tightly controlled (**Figures 6** **, S27–S28**). Not surprisingly, the variation observed is somewhat higher when the bacteria were grown in the complex media, BHI, likely due to variation in the growth conditions and not the array (**Figures S29–S30**).

These results establish that the methods developed to obtain transcriptional data from the *H. influenzae* SGH arrays are consistent and robust within and across chips as well as among biological replicates. Used in conjunction with the filtering pipeline described above we have described the establishment of a unique, robust microarray analysis pipeline for *multi-strain* comparisons specific to *Haemophilus influenzae*. We believe this tool could be of great use to others studying this organism and to those studying other organisms that make use of similar supragenome based arrays who may not have the resources to perform RNAseq or the expertise to deal with the associated downstream bioinformatic data analysis required.

## Supporting Information

Figure S1 **Comparison of probe replicate values within RNA from condition 1, replicate A.** Log_2_ expression values from the two probe sets in condition 1, replicate A on two separate chips (left/right).
(TIF)

Figure S2 **Comparison of probe replicate values within RNA from condition 1, replicate B.** Log_2_ expression values from the two probe sets in condition 1, replicate B on two separate chips (left/right).
(TIF)

Figure S3 **Comparison of probe replicate values within RNA from condition 2, replicate A.** Log_2_ expression values from the two probe sets in condition 2, replicate A on two separate chips (left/right).
(TIF)

Figure S4 **Comparison of probe replicate values within RNA from condition 2, replicate B.** Log_2_ expression values from the two probe sets in condition 2, replicate B on two separate chips (left/right).
(TIF)

Figure S5 **Comparison of probe replicate values within RNA from condition 4, replicate A.** Log_2_ expression values from the two probe sets in condition 4, replicate A on two separate chips (left/right).
(TIF)

Figure S6 **Comparison of probe replicate values within RNA from condition 4, replicate B.** Log_2_ expression values from the two probe sets in condition 4, replicate B on two separate chips (left/right).
(TIF)

Figure S7 **Comparison of probe replicate values within RNA from condition 5, replicate A.** Log_2_ expression values from the two probe sets in condition 5, replicate A on two separate chips (left/right).
(TIF)

Figure S8 **Comparison of probe replicate values within RNA from condition 5, replicate B.** Log_2_ expression values from the two probe sets in condition 5, replicate B on two separate chips (left/right).
(TIF)

Figure S9 **Comparison of probe replicate values within RNA from condition 6, replicate A.** Log_2_ expression values from the two probe sets in condition 6, replicate A on two separate chips (left/right).
(TIF)

Figure S10 **Comparison of probe replicate values within RNA from condition 6, replicate B.** Log_2_ expression values from the two probe sets in condition 6, replicate B on two separate chips (left/right).
(TIF)

Figure S11 **Comparison of probe replicate values within RNA from condition 7, replicate A.** Log_2_ expression values from the two probe sets in condition 7, replicate A on two separate chips (left/right).
(TIF)

Figure S12 **Comparison of probe replicate values within RNA from condition 7, replicate B.** Log_2_ expression values from the two probe sets in condition 7, replicate B on two separate chips (left/right).
(TIF)

Figure S13 **Comparison of probe replicate values within RNA from condition 8, replicate A.** Log_2_ expression values from the two probe sets in condition 8, replicate A on two separate chips (left/right).
(TIF)

Figure S14 **Comparison of probe replicate values within RNA from condition 8, replicate B.** Log_2_ expression values from the two probe sets in condition 8, replicate B on two separate chips (left/right).
(TIF)

Figure S15 **Comparison of probe replicate values within RNA from condition 9, replicate A.** Log_2_ expression values from the two probe sets in condition 9, replicate A on two separate chips (left/right).
(TIF)

Figure S16 **Comparison of probe replicate values within RNA from condition 9, replicate B.** Log_2_ expression values from the two probe sets in condition 9, replicate B on two separate chips (left/right).
(TIF)

Figure S17 **Comparison of probe replicate values within RNA from condition 10, replicate A.** Log_2_ expression values from the two probe sets in condition 10, replicate A on two separate chips (left/right).
(TIF)

Figure S18 **Comparison of probe replicate values within RNA from condition 10, replicate B.** Log_2_ expression values from the two probe sets in condition 10, replicate B on two separate chips (left/right).
(TIF)

Figure S19 **Comparison of chip replicate values for RNA from condition 1.** Log_2_ expression values (average of two probe-sets) from the same RNA samples run on two different chips: chip1 (x-axes) and chip2 (y-axes). Condition 1, replicate A (left) and replicate B (right).
(TIF)

Figure S20 **Comparison of chip replicate values for RNA from condition 2.** Log_2_ expression values (average of two probe-sets) from the same RNA samples run on two different chips: chip1 (x-axes) and chip2 (y-axes). Condition 2, replicate A (left) and replicate B (right).
(TIF)

Figure S21 **Comparison of chip replicate values for RNA from condition 5.** Log_2_ expression values (average of two probe-sets) from the same RNA samples run on two different chips: chip1 (x-axes) and chip2 (y-axes). Condition 5, replicate A (left) and replicate B (right).
(TIF)

Figure S22 **Comparison of chip replicate values for RNA from condition 6.** Log_2_ expression values (average of two probe-sets) from the same RNA samples run on two different chips: chip1 (x-axes) and chip2 (y-axes). Condition 6, replicate A (left) and replicate B (right).
(TIF)

Figure S23 **Comparison of chip replicate values for RNA from condition 7.** Log_2_ expression values (average of two probe-sets) from the same RNA samples run on two different chips: chip1 (x-axes) and chip2 (y-axes). Condition 7, replicate A (left) and replicate B (right).
(TIF)

Figure S24 **Comparison of chip replicate values for RNA from condition 8.** Log_2_ expression values (average of two probe-sets) from the same RNA samples run on two different chips: chip1 (x-axes) and chip2 (y-axes). Condition 8, replicate A (left) and replicate B (right).
(TIF)

Figure S25 **Comparison of chip replicate values for RNA from condition 9.** Log_2_ expression values (average of two probe-sets) from the same RNA samples run on two different chips: chip1 (x-axes) and chip2 (y-axes). Condition 9, replicate A (left) and replicate B (right).
(TIF)

Figure S26 **Comparison of chip replicate values for RNA from condition 10.** Log_2_ expression values (average of two probe-sets) from the same RNA samples run on two different chips: chip1 (x-axes) and chip2 (y-axes). Condition 10, replicate A (left) and replicate B (right).
(TIF)

Figure S27 **Biological replicate comparison from Condition 1 and 2.** Log_2_ expression values (averages of both probe-sets from both chips) from the two biological replicate samples for both condition 1 (left) and condition 2 (right).
(TIF)

Figure S28 **Biological replicate comparison from Condition 5 and 6.** Log_2_ expression values (averages of both probe-sets from both chips) from the two biological replicate samples for both condition 5 (left) and condition 6 (right).
(TIF)

Figure S29 **Biological replicate comparison from Condition 7 and 8.** Log_2_ expression values (averages of both probe-sets from both chips) from the two biological replicate samples for both condition 7 (left) and condition 8 (right).
(TIF)

Figure S30 **Biological replicate comparison from Condition 9 and 10.** Log_2_ expression values (averages of both probe-sets from both chips) from the two biological replicate samples for both condition 9 (left) and condition 10 (right).
(TIF)

Table S1 **Source of RNA for each “condition”.**
(DOCX)

Table S2 **False positives found using a p-value<0.05 cutoff.** Condition 4, replicate B, chip 1 and chip 2 were compared. Raw expression values are shown. FDR: False discovery rate, BH: Benjamini-Hochberg, Bon. pVal: Bonferroni-corrected p-value.
(DOCX)

Table S3 **False positives using a fold change cutoff of >1.5.** Condition 4, replicate B, chip 1 and chip 2 were compared. Raw expression values are shown. FDR: False discovery rate, BH: Benjamini-Hochberg, Bon. pVal: Bonferroni-corrected p-value.
(DOCX)
