# Supplementary figures and images for: Development and Validation of an Haemophilus influenzae Supragenome Hybridization (SGH) Array for Transcriptomic Analyses
Source: PLoS One. 2014 Oct 7;9(10):e105493. doi: 10.1371/journal.pone.0105493 (PMC4188559; doi:10.1371/journal.pone.0105493)

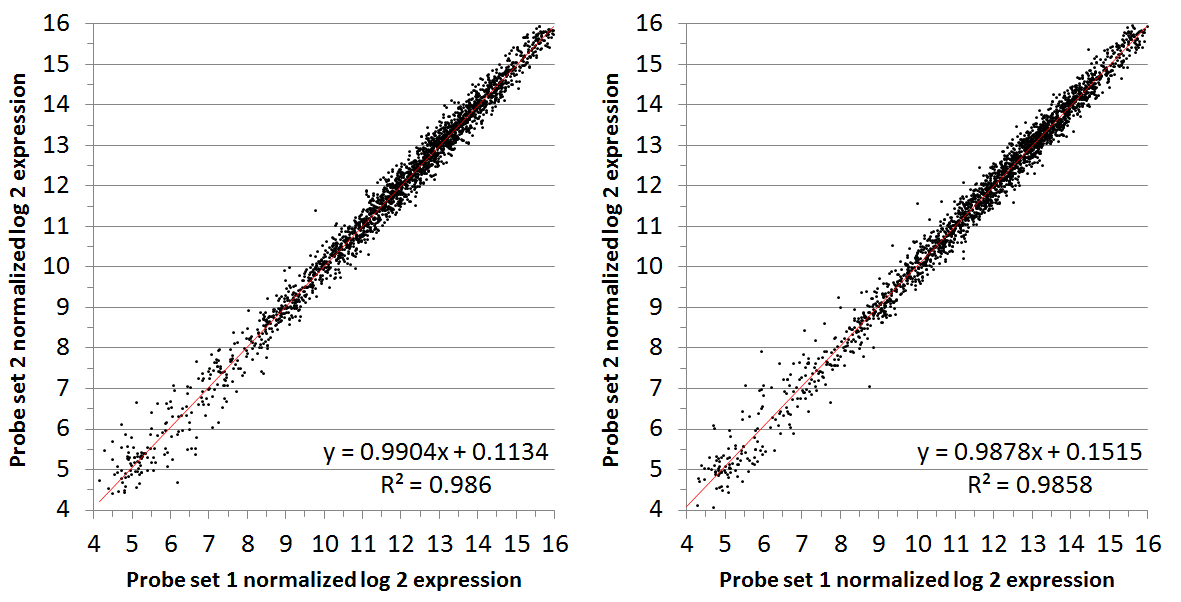

Supplement: Figure S1 — Comparison of probe replicate values within RNA from condition 1, replicate A. Log2 expression values from the two probe sets in condition 1, replicate A on two separate chips (left/right). (TIF) [file pone.0105493.s001.tif]

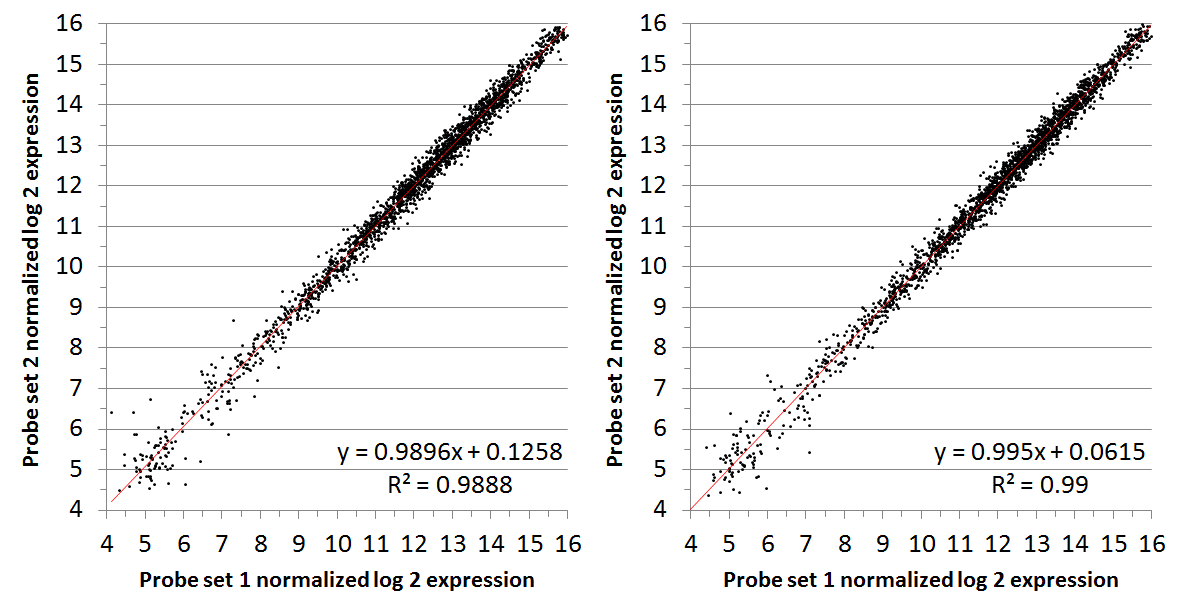

Supplement: Figure S2 — Comparison of probe replicate values within RNA from condition 1, replicate B. Log2 expression values from the two probe sets in condition 1, replicate B on two separate chips (left/right). (TIF) [file pone.0105493.s002.tif]

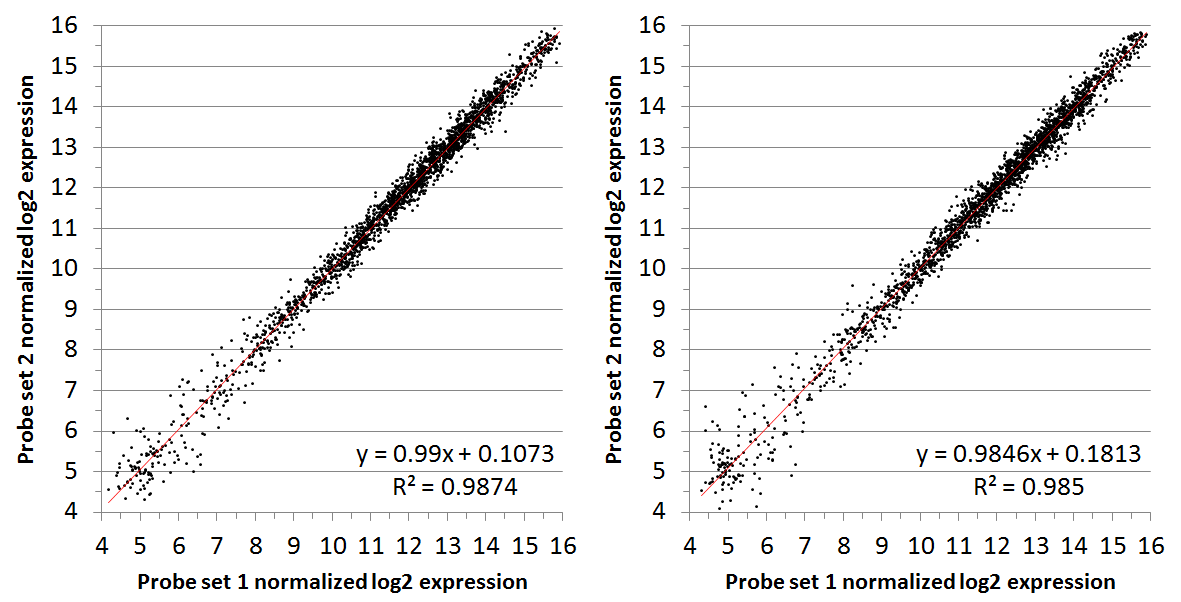

Supplement: Figure S3 — Comparison of probe replicate values within RNA from condition 2, replicate A. Log2 expression values from the two probe sets in condition 2, replicate A on two separate chips (left/right). (TIF) [file pone.0105493.s003.tif]

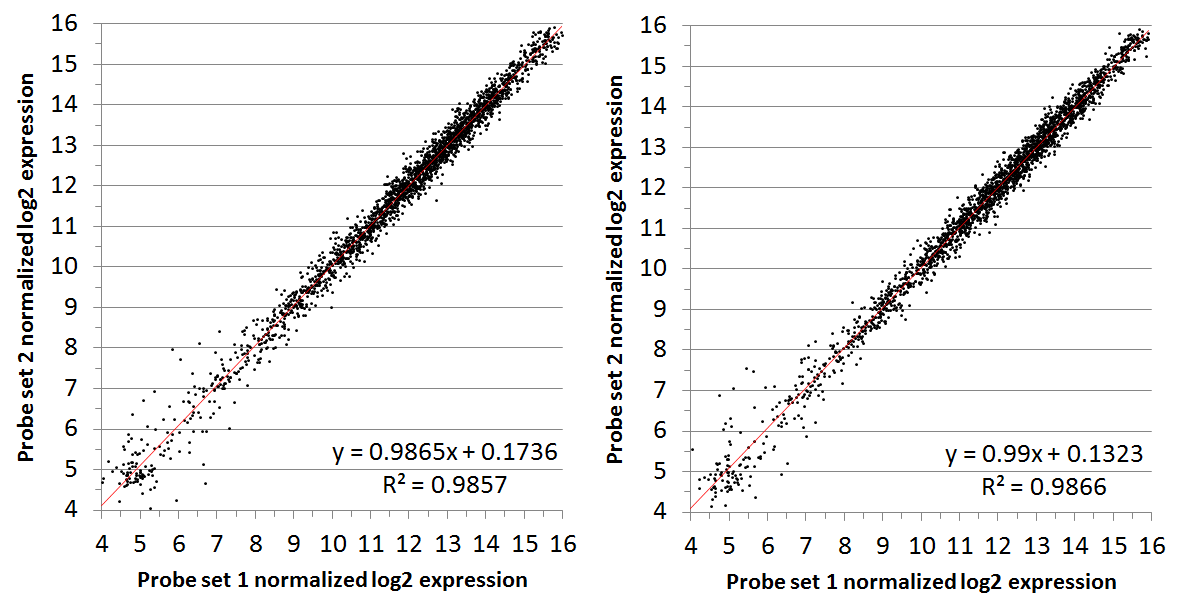

Supplement: Figure S4 — Comparison of probe replicate values within RNA from condition 2, replicate B. Log2 expression values from the two probe sets in condition 2, replicate B on two separate chips (left/right). (TIF) [file pone.0105493.s004.tif]

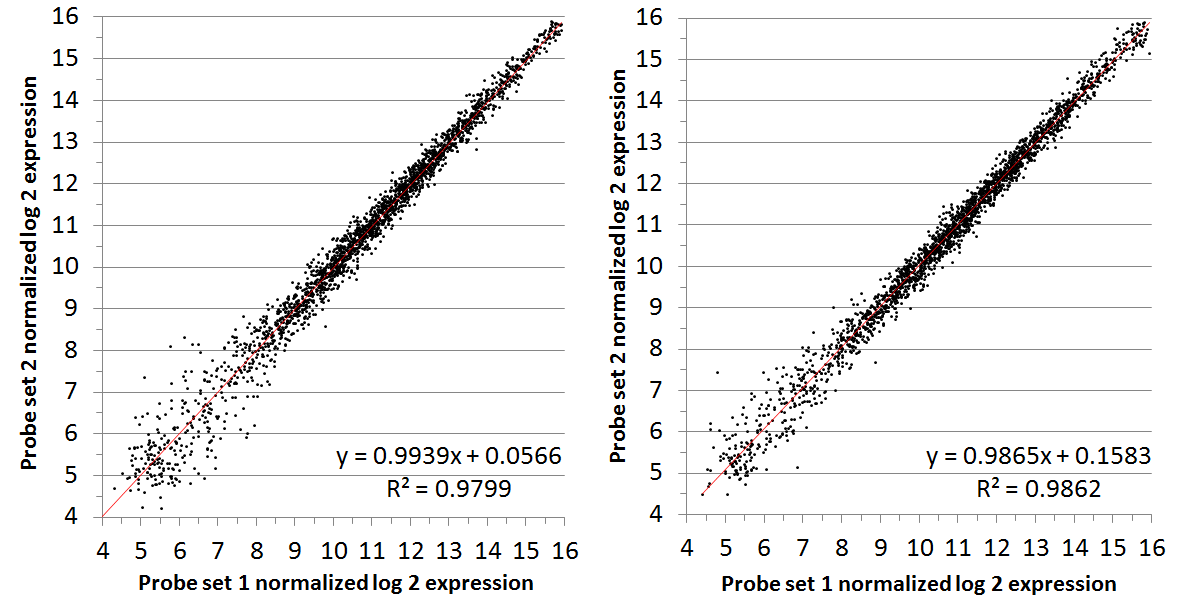

Supplement: Figure S5 — Comparison of probe replicate values within RNA from condition 4, replicate A. Log2 expression values from the two probe sets in condition 4, replicate A on two separate chips (left/right). (TIF) [file pone.0105493.s005.tif]

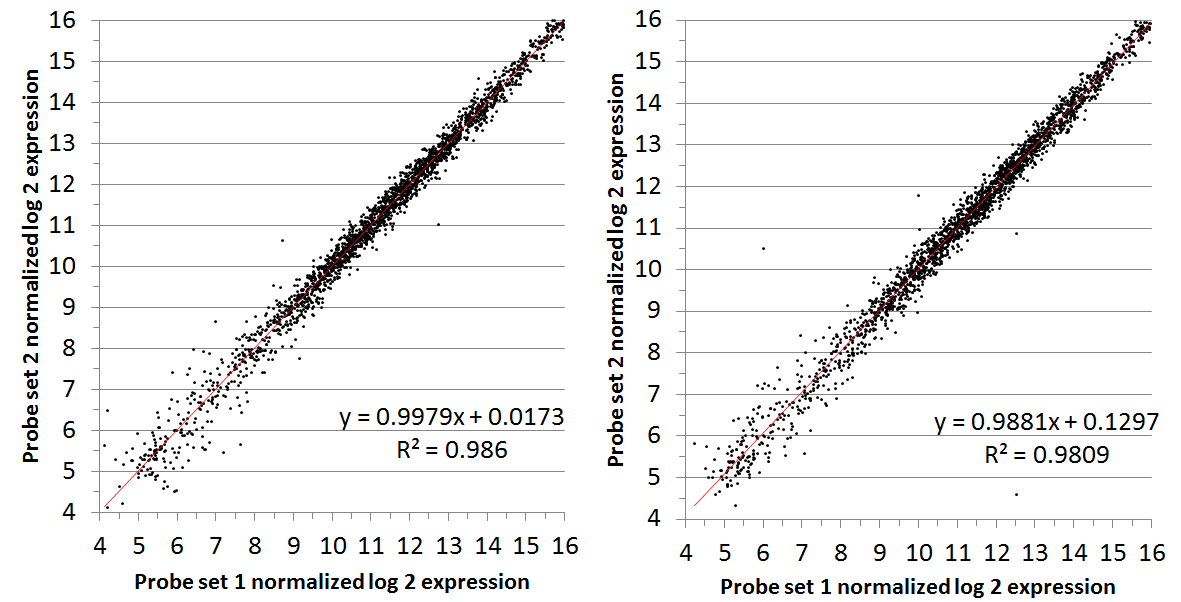

Supplement: Figure S6 — Comparison of probe replicate values within RNA from condition 4, replicate B. Log2 expression values from the two probe sets in condition 4, replicate B on two separate chips (left/right). (TIF) [file pone.0105493.s006.tif]

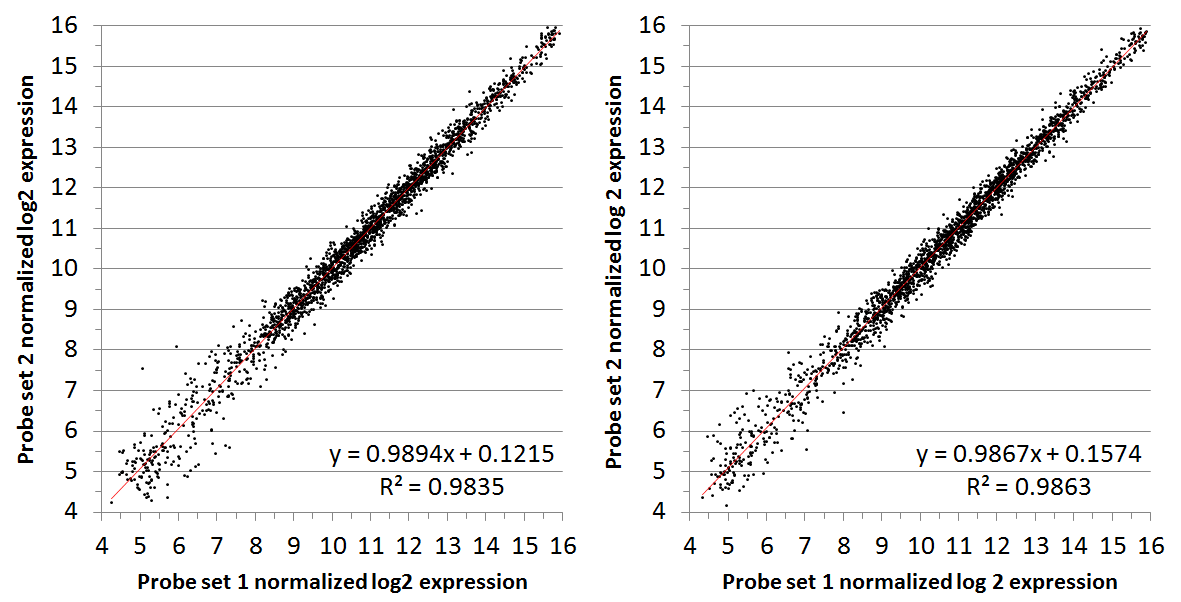

Supplement: Figure S7 — Comparison of probe replicate values within RNA from condition 5, replicate A. Log2 expression values from the two probe sets in condition 5, replicate A on two separate chips (left/right). (TIF) [file pone.0105493.s007.tif]

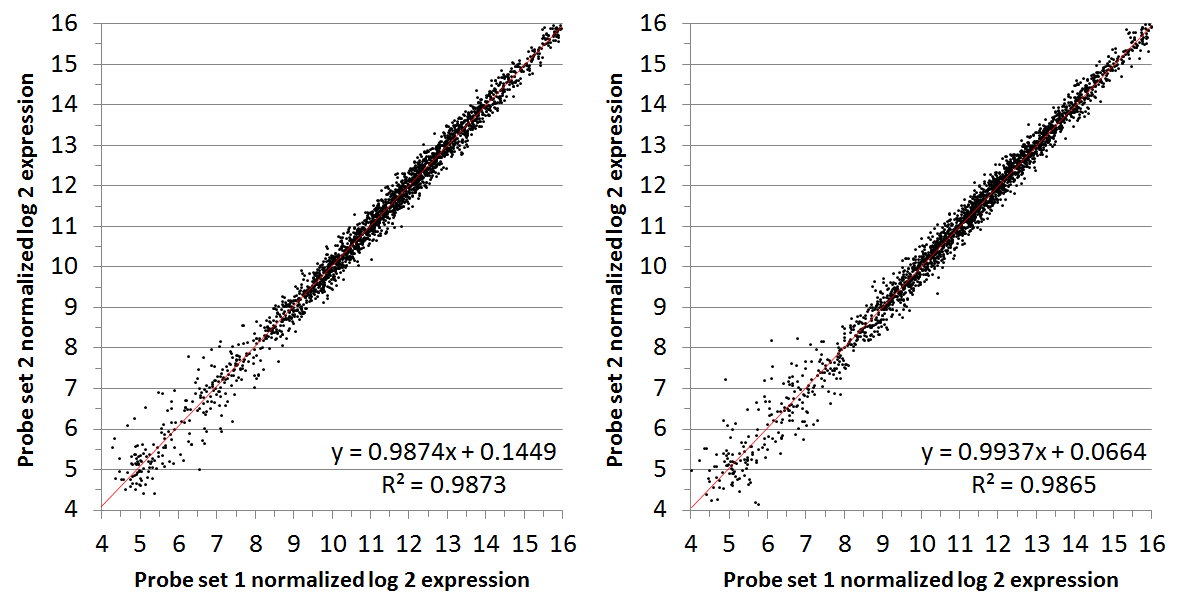

Supplement: Figure S8 — Comparison of probe replicate values within RNA from condition 5, replicate B. Log2 expression values from the two probe sets in condition 5, replicate B on two separate chips (left/right). (TIF) [file pone.0105493.s008.tif]

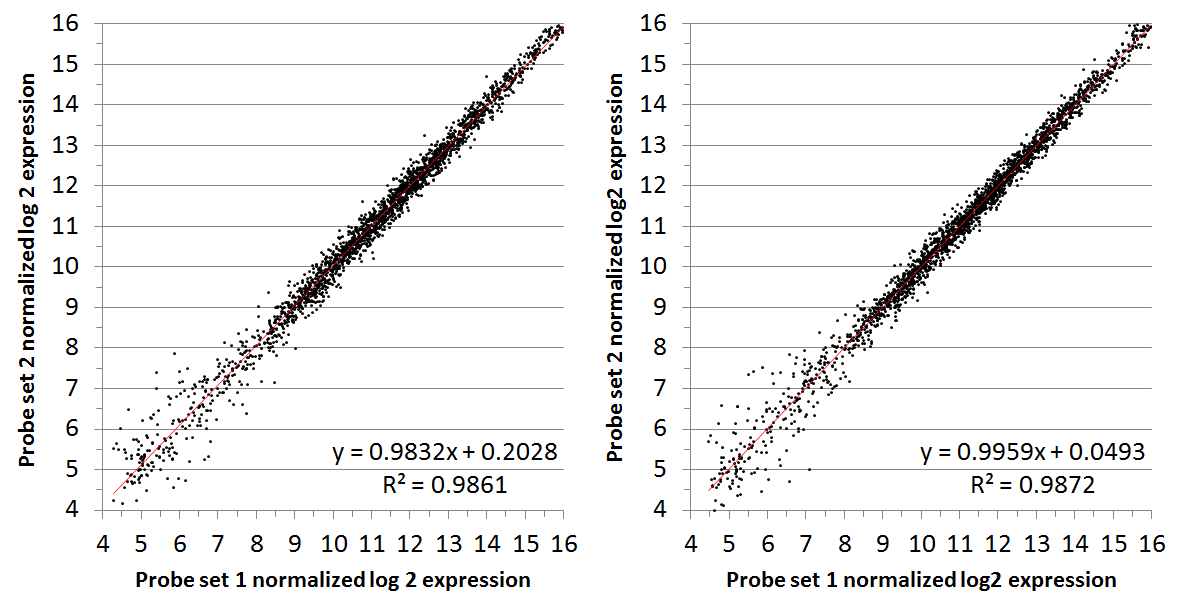

Supplement: Figure S9 — Comparison of probe replicate values within RNA from condition 6, replicate A. Log2 expression values from the two probe sets in condition 6, replicate A on two separate chips (left/right). (TIF) [file pone.0105493.s009.tif]

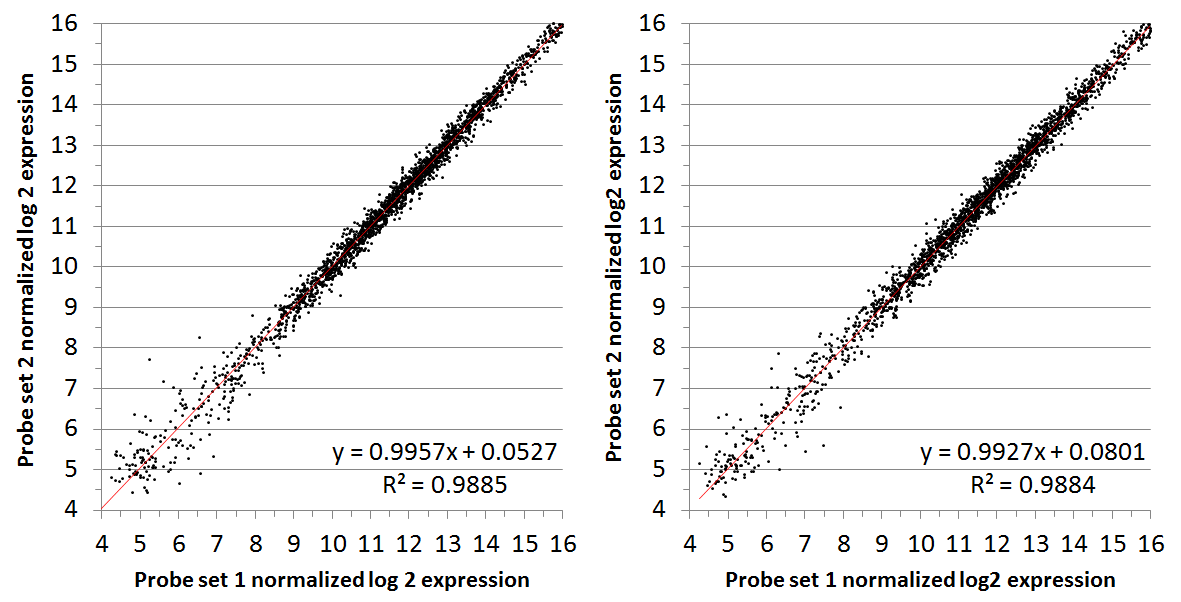

Supplement: Figure S10 — Comparison of probe replicate values within RNA from condition 6, replicate B. Log2 expression values from the two probe sets in condition 6, replicate B on two separate chips (left/right). (TIF) [file pone.0105493.s010.tif]

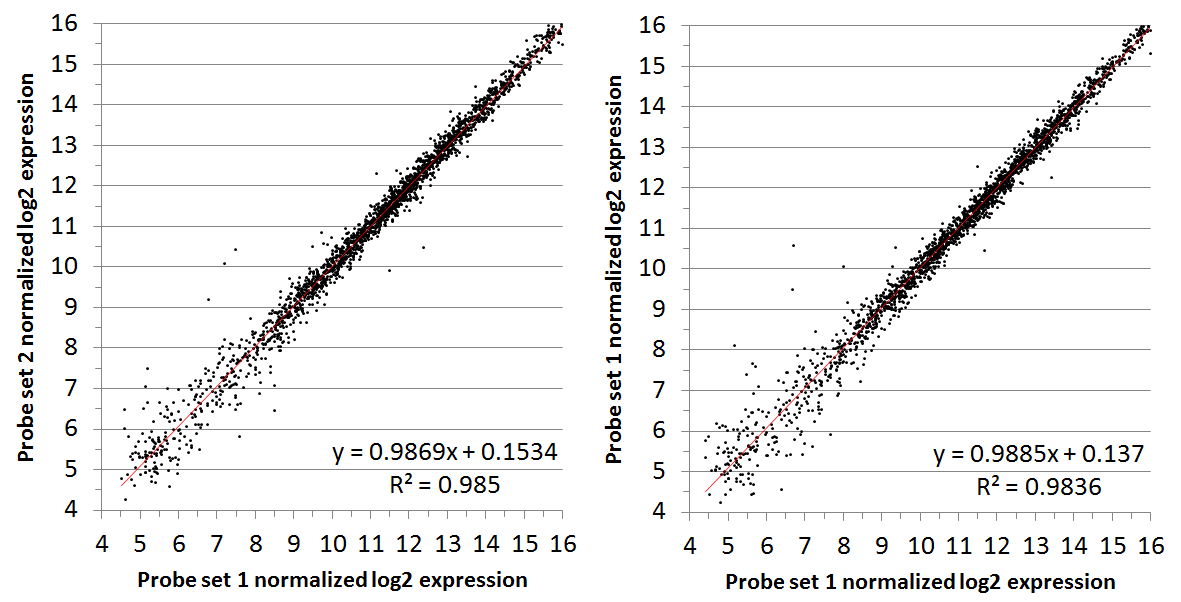

Supplement: Figure S11 — Comparison of probe replicate values within RNA from condition 7, replicate A. Log2 expression values from the two probe sets in condition 7, replicate A on two separate chips (left/right). (TIF) [file pone.0105493.s011.tif]

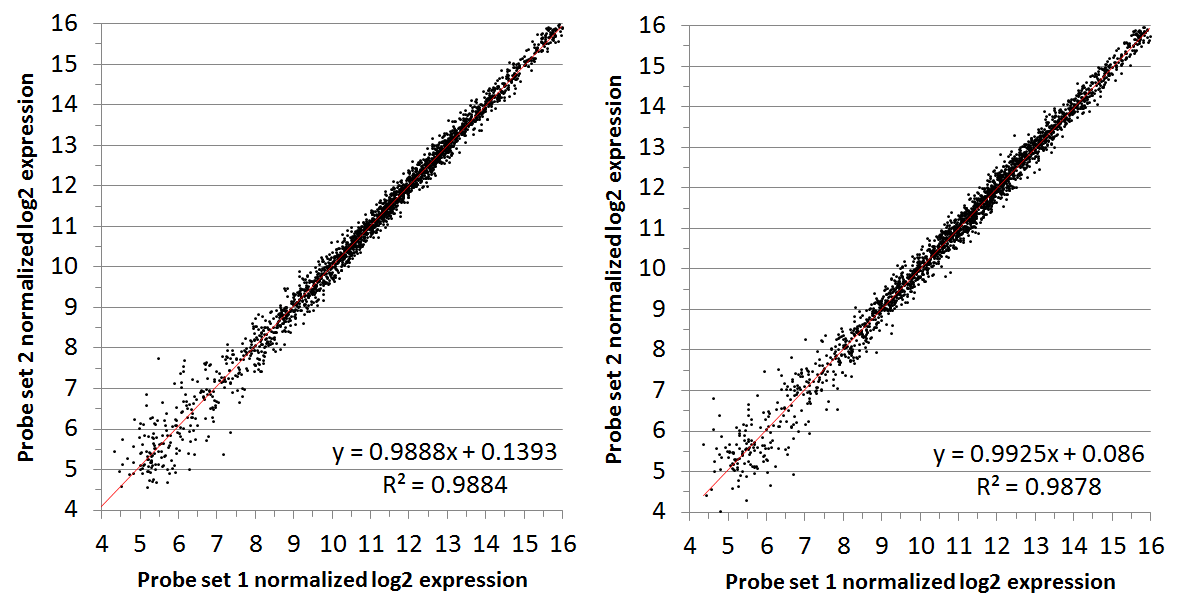

Supplement: Figure S12 — Comparison of probe replicate values within RNA from condition 7, replicate B. Log2 expression values from the two probe sets in condition 7, replicate B on two separate chips (left/right). (TIF) [file pone.0105493.s012.tif]

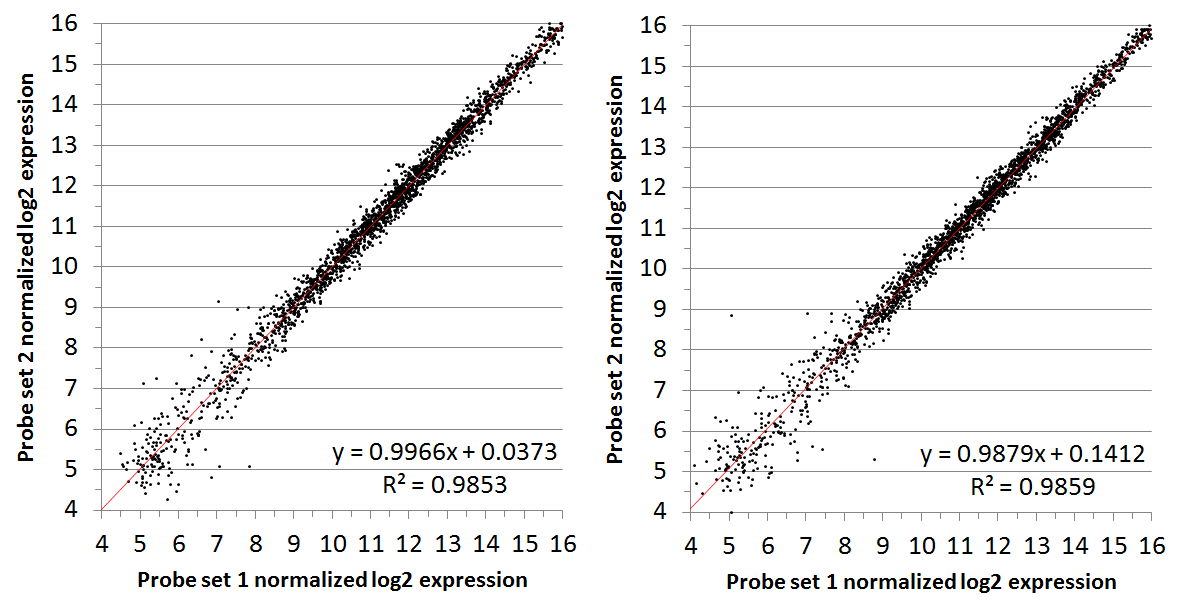

Supplement: Figure S13 — Comparison of probe replicate values within RNA from condition 8, replicate A. Log2 expression values from the two probe sets in condition 8, replicate A on two separate chips (left/right). (TIF) [file pone.0105493.s013.tif]

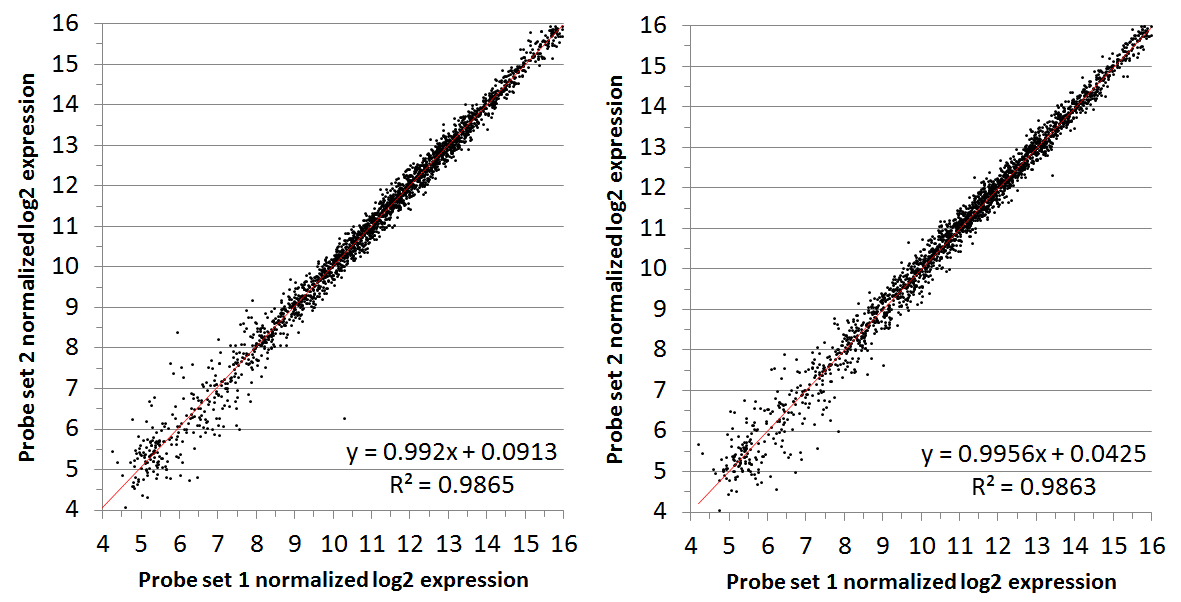

Supplement: Figure S14 — Comparison of probe replicate values within RNA from condition 8, replicate B. Log2 expression values from the two probe sets in condition 8, replicate B on two separate chips (left/right). (TIF) [file pone.0105493.s014.tif]

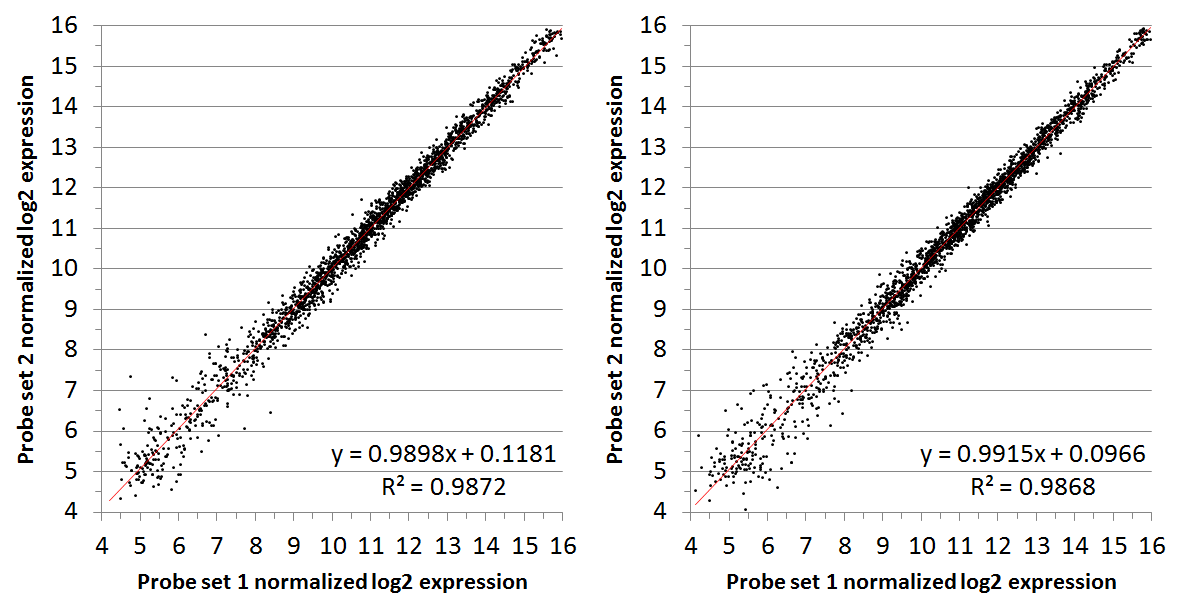

Supplement: Figure S15 — Comparison of probe replicate values within RNA from condition 9, replicate A. Log2 expression values from the two probe sets in condition 9, replicate A on two separate chips (left/right). (TIF) [file pone.0105493.s015.tif]

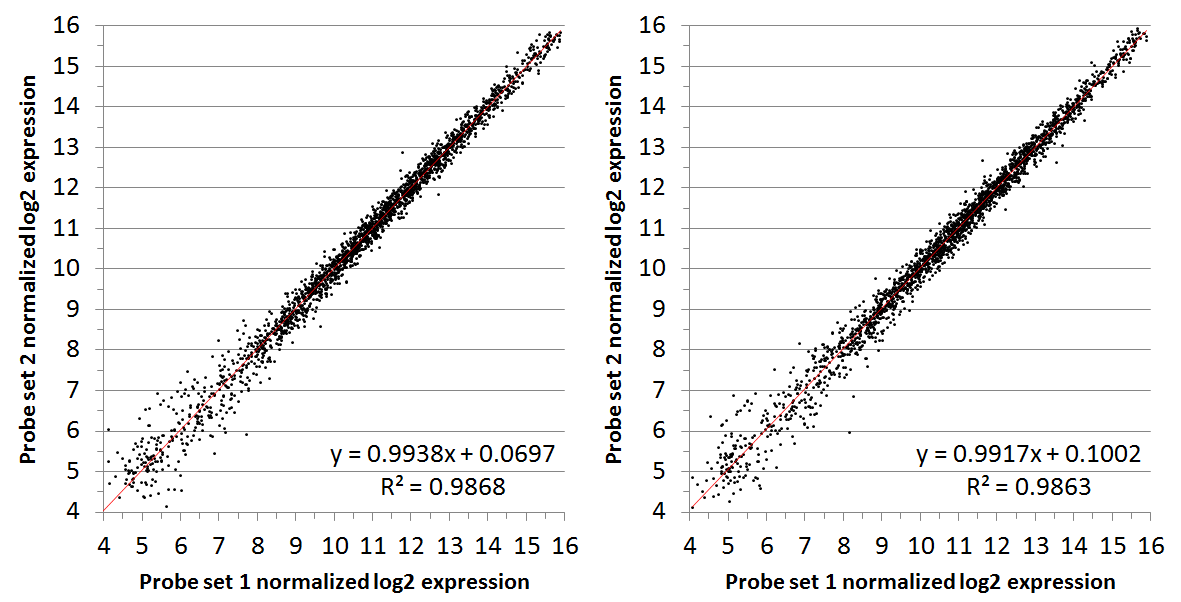

Supplement: Figure S16 — Comparison of probe replicate values within RNA from condition 9, replicate B. Log2 expression values from the two probe sets in condition 9, replicate B on two separate chips (left/right). (TIF) [file pone.0105493.s016.tif]

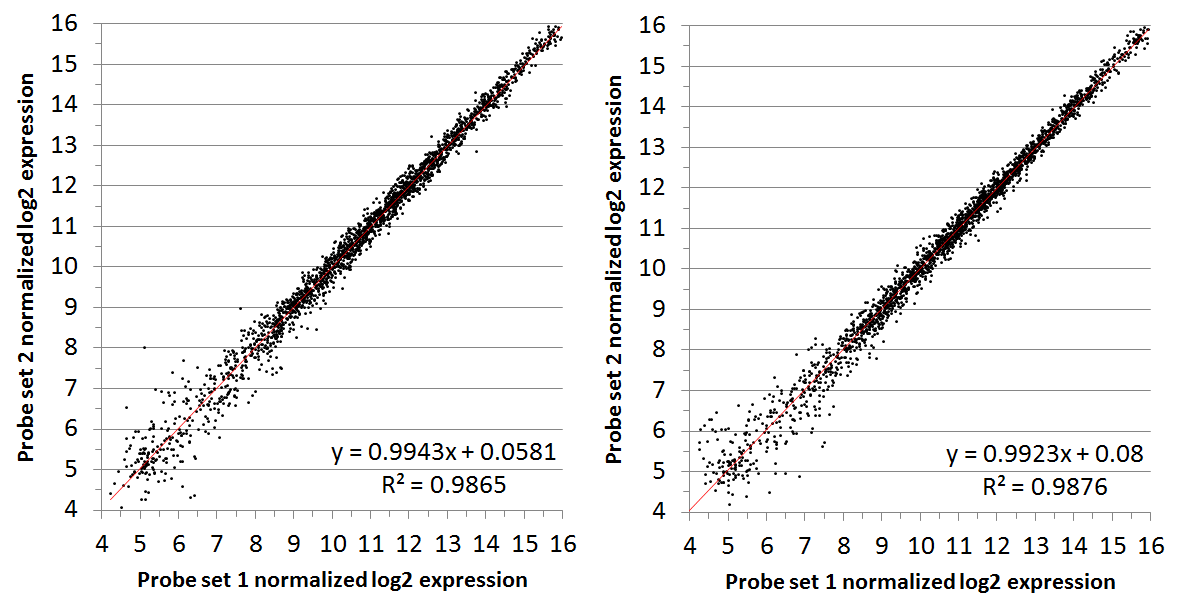

Supplement: Figure S17 — Comparison of probe replicate values within RNA from condition 10, replicate A. Log2 expression values from the two probe sets in condition 10, replicate A on two separate chips (left/right). (TIF) [file pone.0105493.s017.tif]

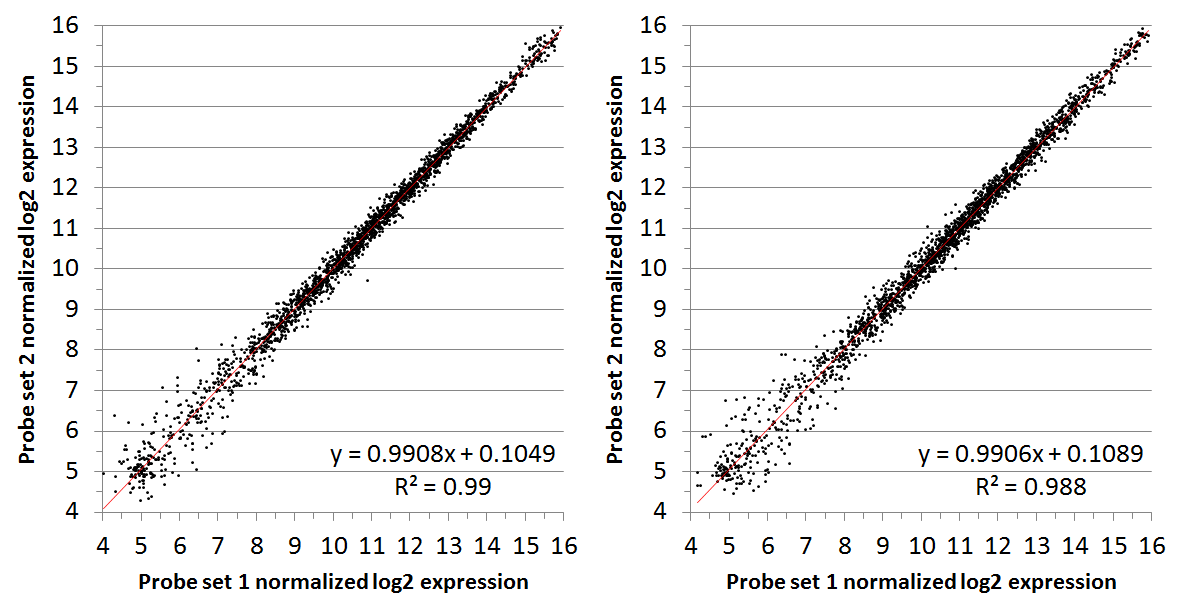

Supplement: Figure S18 — Comparison of probe replicate values within RNA from condition 10, replicate B. Log2 expression values from the two probe sets in condition 10, replicate B on two separate chips (left/right). (TIF) [file pone.0105493.s018.tif]

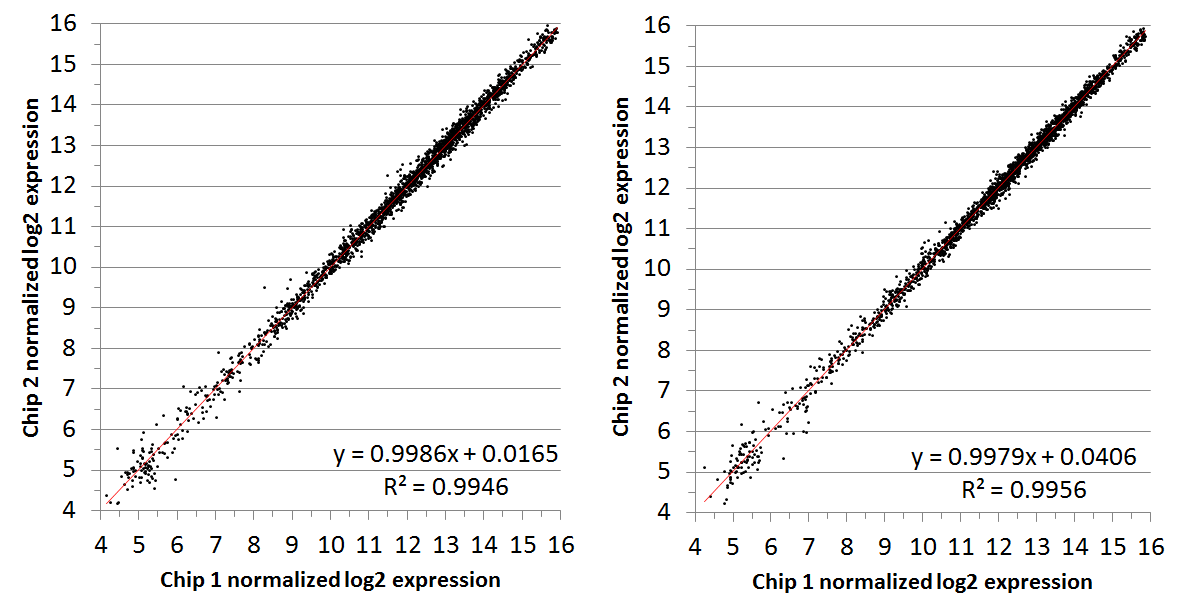

Supplement: Figure S19 — Comparison of chip replicate values for RNA from condition 1. Log2 expression values (average of two probe-sets) from the same RNA samples run on two different chips: chip1 (x-axes) and chip2 (y-axes). Condition 1, replicate A (left) and replicate B (right). (TIF) [file pone.0105493.s019.tif]

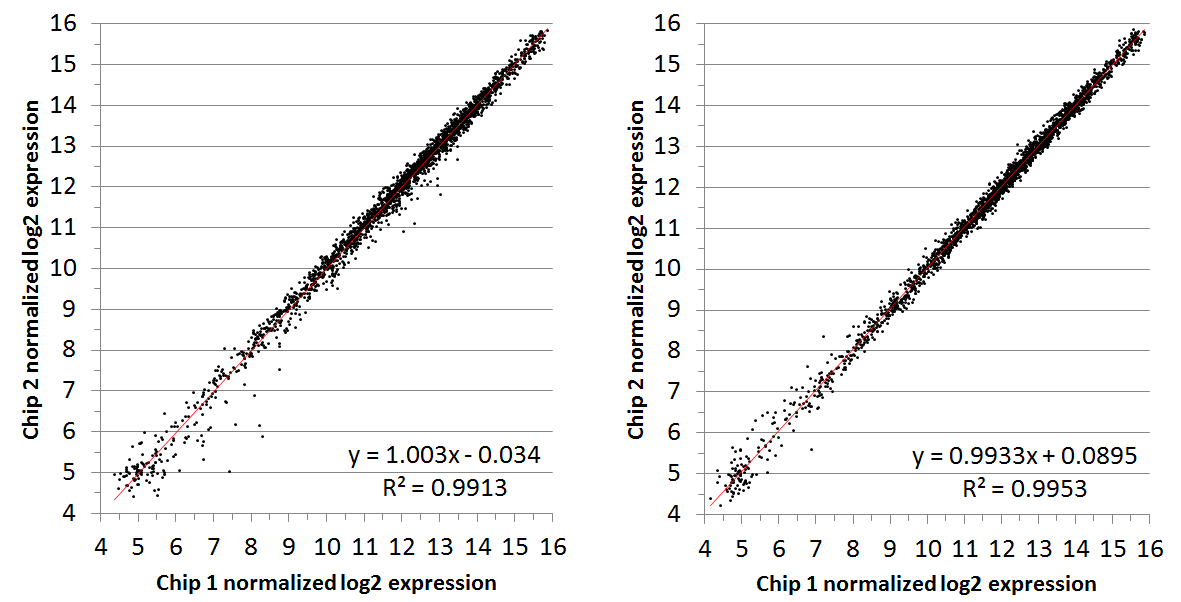

Supplement: Figure S20 — Comparison of chip replicate values for RNA from condition 2. Log2 expression values (average of two probe-sets) from the same RNA samples run on two different chips: chip1 (x-axes) and chip2 (y-axes). Condition 2, replicate A (left) and replicate B (right). (TIF) [file pone.0105493.s020.tif]

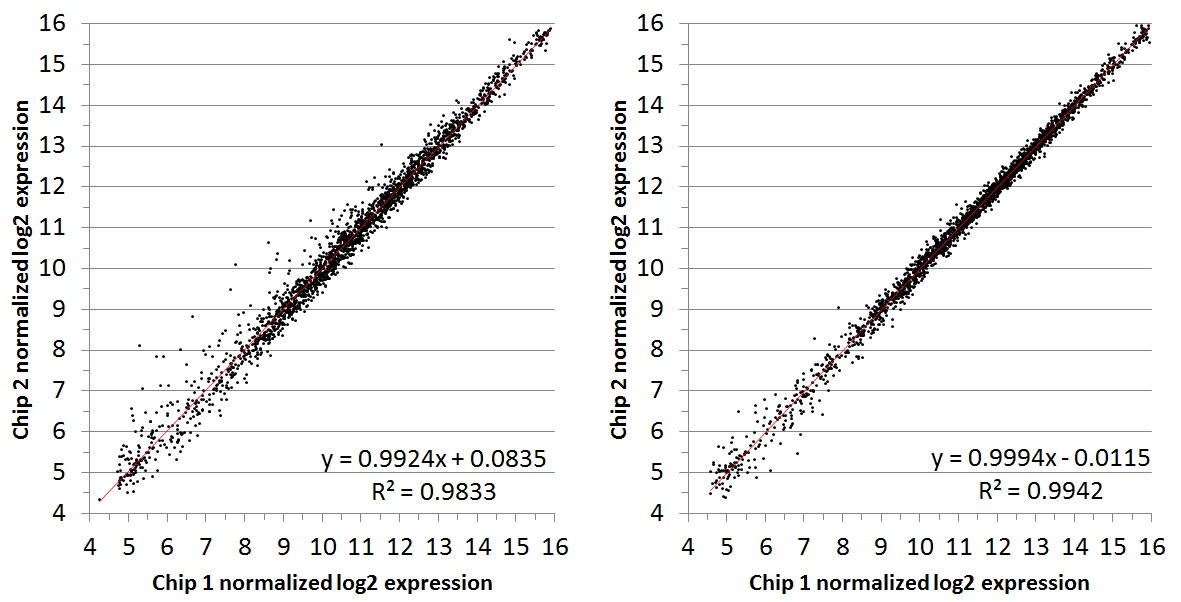

Supplement: Figure S21 — Comparison of chip replicate values for RNA from condition 5. Log2 expression values (average of two probe-sets) from the same RNA samples run on two different chips: chip1 (x-axes) and chip2 (y-axes). Condition 5, replicate A (left) and replicate B (right). (TIF) [file pone.0105493.s021.tif]

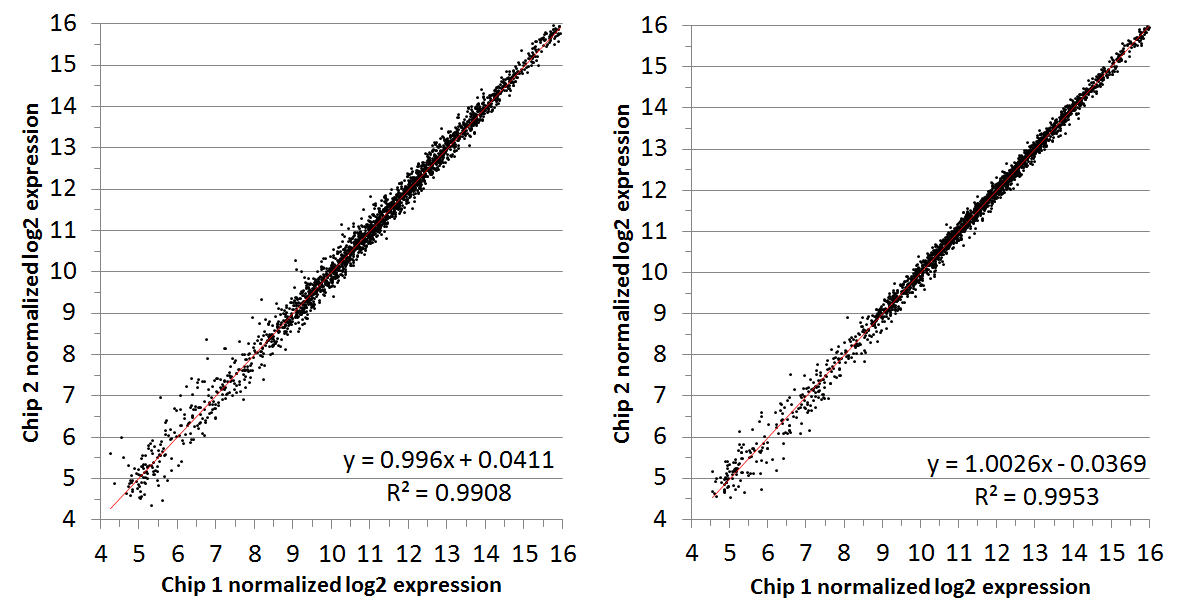

Supplement: Figure S22 — Comparison of chip replicate values for RNA from condition 6. Log2 expression values (average of two probe-sets) from the same RNA samples run on two different chips: chip1 (x-axes) and chip2 (y-axes). Condition 6, replicate A (left) and replicate B (right). (TIF) [file pone.0105493.s022.tif]

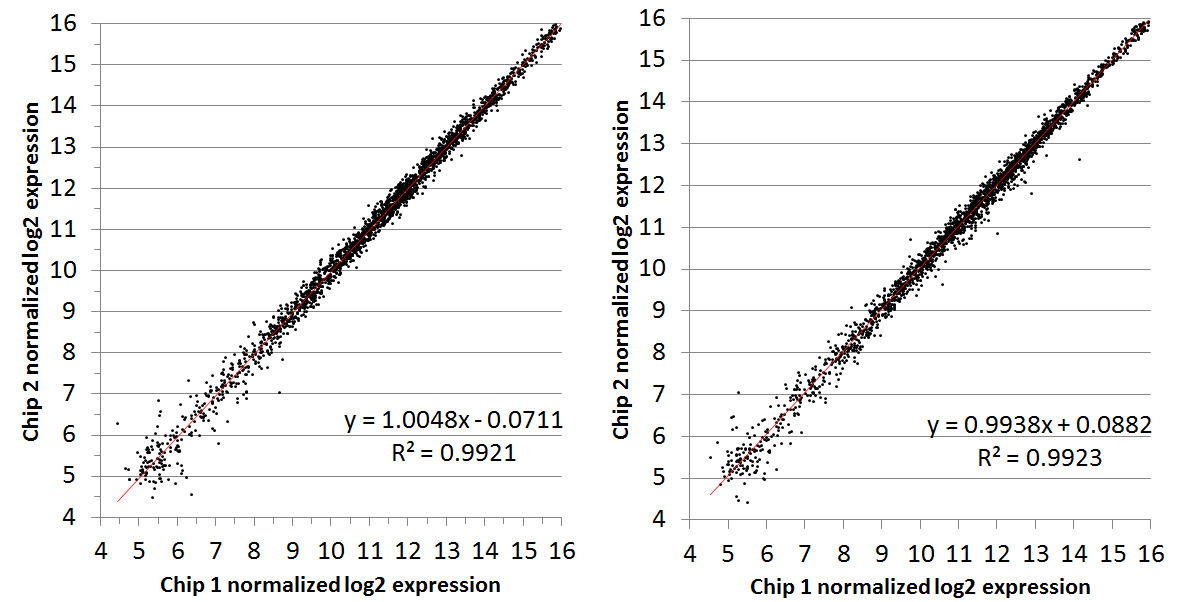

Supplement: Figure S23 — Comparison of chip replicate values for RNA from condition 7. Log2 expression values (average of two probe-sets) from the same RNA samples run on two different chips: chip1 (x-axes) and chip2 (y-axes). Condition 7, replicate A (left) and replicate B (right). (TIF) [file pone.0105493.s023.tif]

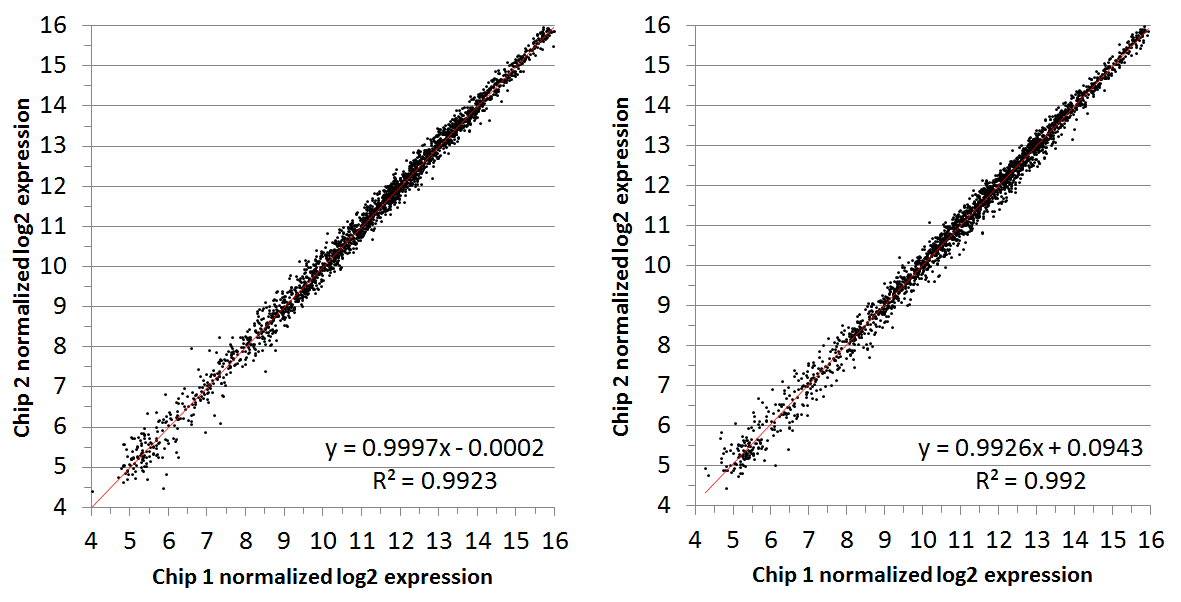

Supplement: Figure S24 — Comparison of chip replicate values for RNA from condition 8. Log2 expression values (average of two probe-sets) from the same RNA samples run on two different chips: chip1 (x-axes) and chip2 (y-axes). Condition 8, replicate A (left) and replicate B (right). (TIF) [file pone.0105493.s024.tif]

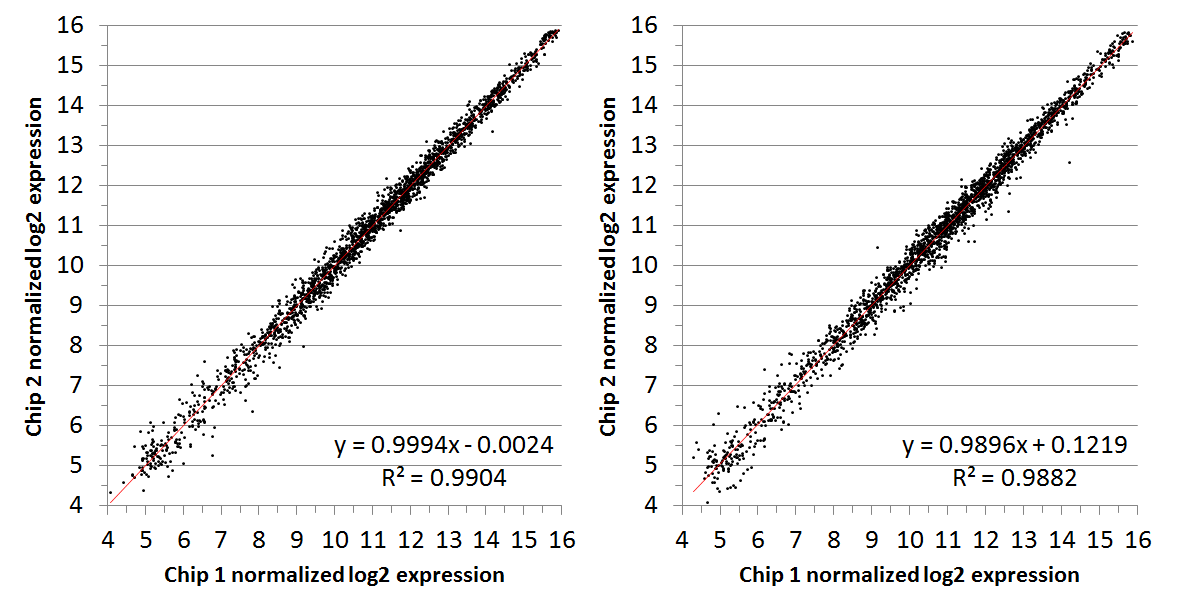

Supplement: Figure S25 — Comparison of chip replicate values for RNA from condition 9. Log2 expression values (average of two probe-sets) from the same RNA samples run on two different chips: chip1 (x-axes) and chip2 (y-axes). Condition 9, replicate A (left) and replicate B (right). (TIF) [file pone.0105493.s025.tif]

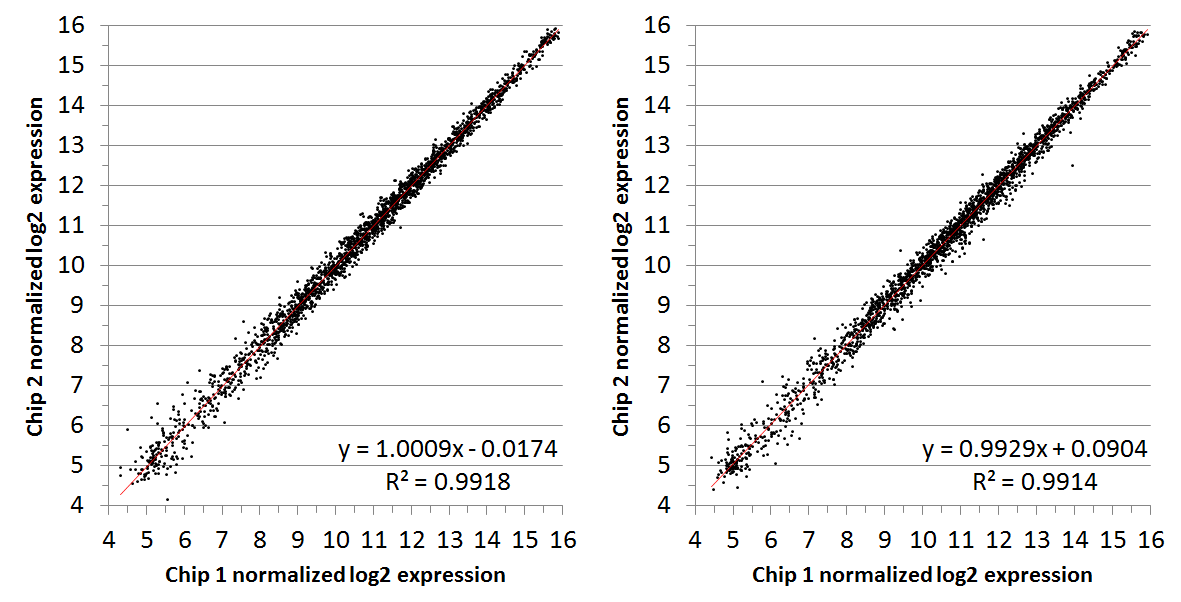

Supplement: Figure S26 — Comparison of chip replicate values for RNA from condition 10. Log2 expression values (average of two probe-sets) from the same RNA samples run on two different chips: chip1 (x-axes) and chip2 (y-axes). Condition 10, replicate A (left) and replicate B (right). (TIF) [file pone.0105493.s026.tif]

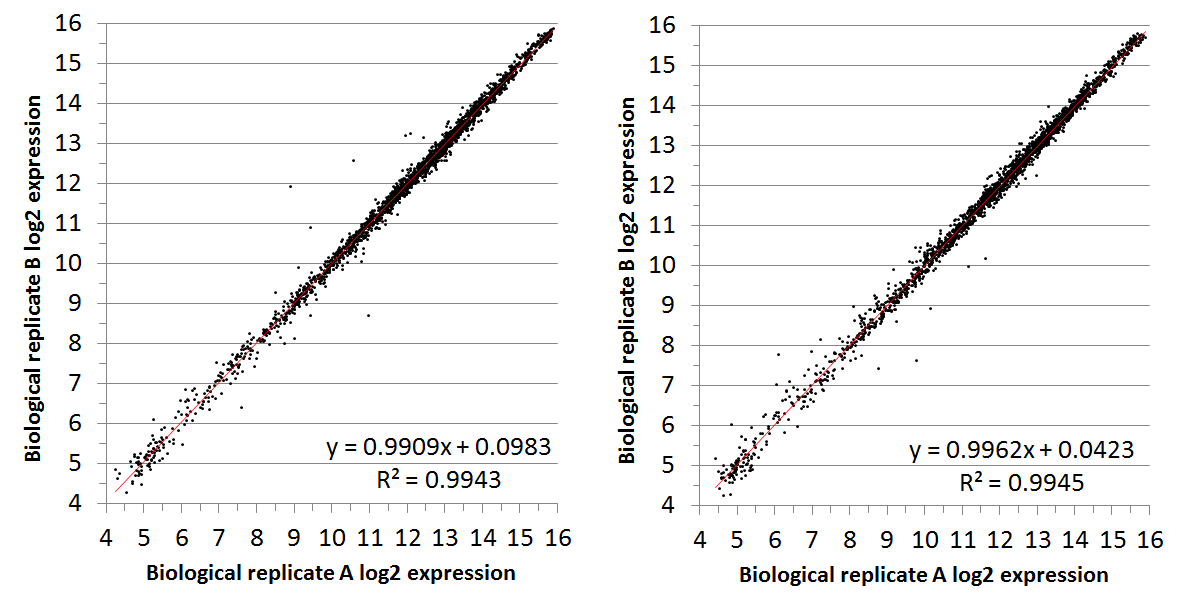

Supplement: Figure S27 — Biological replicate comparison from Condition 1 and 2. Log2 expression values (averages of both probe-sets from both chips) from the two biological replicate samples for both condition 1 (left) and condition 2 (right). (TIF) [file pone.0105493.s027.tif]

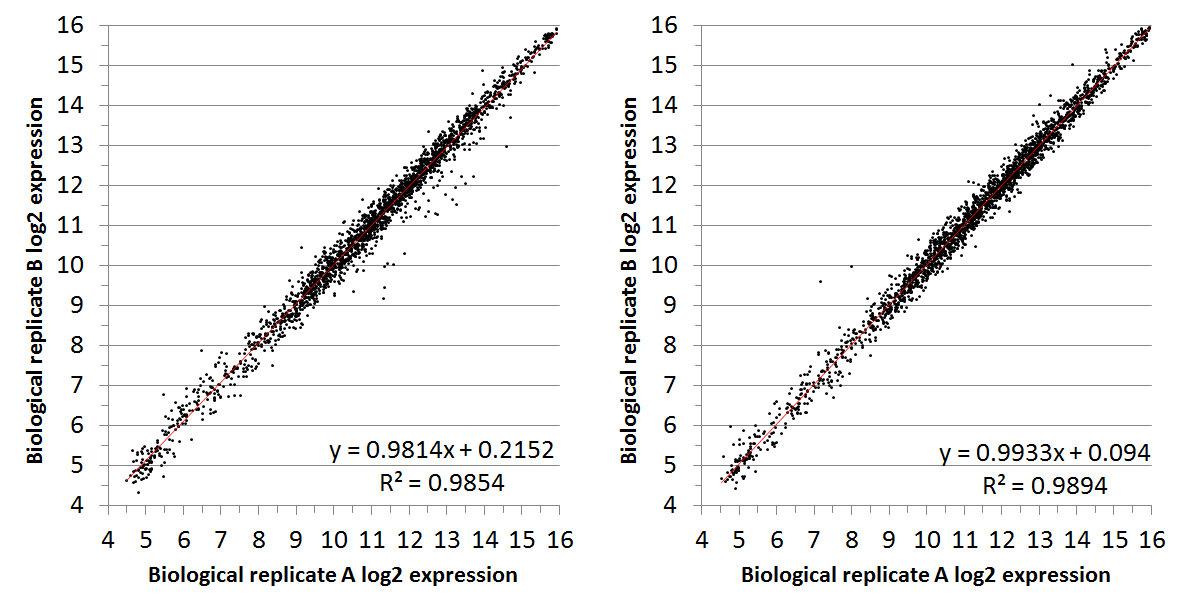

Supplement: Figure S28 — Biological replicate comparison from Condition 5 and 6. Log2 expression values (averages of both probe-sets from both chips) from the two biological replicate samples for both condition 5 (left) and condition 6 (right). (TIF) [file pone.0105493.s028.tif]

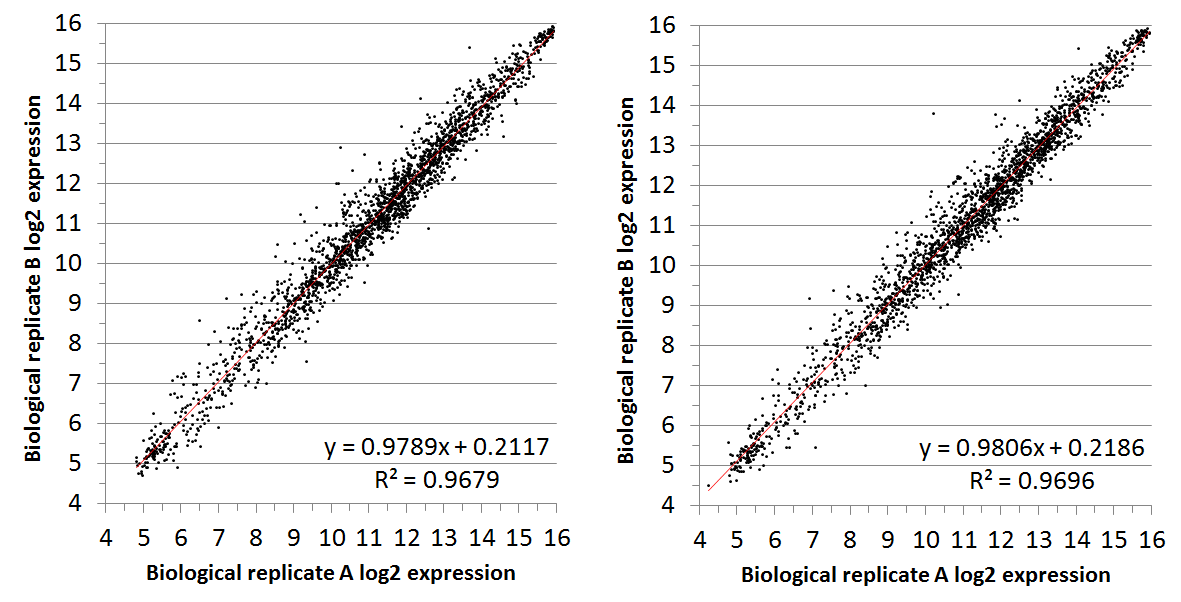

Supplement: Figure S29 — Biological replicate comparison from Condition 7 and 8. Log2 expression values (averages of both probe-sets from both chips) from the two biological replicate samples for both condition 7 (left) and condition 8 (right). (TIF) [file pone.0105493.s029.tif]

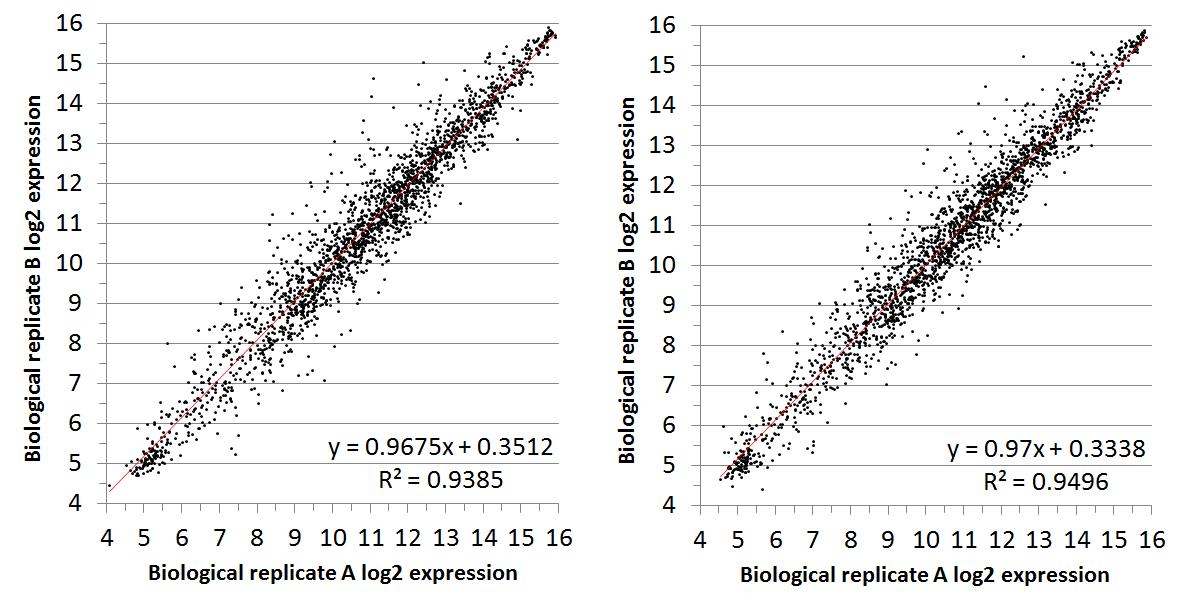

Supplement: Figure S30 — Biological replicate comparison from Condition 9 and 10. Log2 expression values (averages of both probe-sets from both chips) from the two biological replicate samples for both condition 9 (left) and condition 10 (right). (TIF) [file pone.0105493.s030.tif]
