# Supplementary material for: Development and Validation of an Haemophilus influenzae Supragenome Hybridization (SGH) Array for Transcriptomic Analyses
Source: PLoS One. 2014 Oct 7;9(10):e105493. doi: 10.1371/journal.pone.0105493 (PMC4188559; doi:10.1371/journal.pone.0105493)
Supplement: Table S1 — Source of RNA for each “condition”. (DOCX) [file pone.0105493.s031.docx]

**Table S1.** Source of RNA for each “condition”.

| **Condition** | **Description** |
| --- | --- |
| 1 | WT CZ4126/02 grown in in CDM media to OD_A600_ 0.35 |
| 2 | CZ4126/02ΔLsr::Cm^r^ grown in CDM media to OD_A600_ 0.35 |
| 3 | WT CZ4126/02 grown in in CDM media to OD_A600_ 1.0 |
| 4 | CZ4126/02ΔLsr::Cm^r^ grown in CDM media to OD_A600_ 1.0 |
| 5 | WT CZ4126/02 grown in in CDM media to OD_A600_ 1.1 |
| 6 | CZ4126/02ΔLsr::Cm^r^ grown in CDM media to OD_A600_ 1.1 |
| 7 | WT CZ4126/02 grown in in BHI media to OD_A600_ 1.0 |
| 8 | CZ4126/02ΔLsr::Cm^r^ grown in BHI media to OD_A600_ 1.0 |
| 9 | WT CZ4126/02 grown in in BHI media to OD_A600_ 1.1 |
| 10 | CZ4126/02ΔLsr::Cm^r^ grown in BHI media to OD_A600_ 1.1 |
