# Supplementary material for: Development and Validation of an Haemophilus influenzae Supragenome Hybridization (SGH) Array for Transcriptomic Analyses
Source: PLoS One. 2014 Oct 7;9(10):e105493. doi: 10.1371/journal.pone.0105493 (PMC4188559; doi:10.1371/journal.pone.0105493)
Supplement: Table S2 — False positives found using a p-value<0.05 cutoff. Condition 4, replicate B, chip 1 and chip 2 were compared. Raw expression values are shown. FDR: False discovery rate, BH: Benjamini-Hochberg, Bon. pVal: Bonferroni-corrected p-value. (DOCX) [file pone.0105493.s032.docx]

Table S2. False positives found using a p-value < 0.05 cutoff

Condition 4, replicate B, chip 1 and chip 2 were compared. Raw expression values are shown. FDR: False discovery rate, BH: Benjamini-Hochberg, Bon. pVal: Bonferroni-corrected p-value

| **Subcluster** | **Chip1 expression** | **Chip 2 expression** | **FOLD** | **SAM FDR (%)** | **pVal** | **BH FDR** | **Bon. pVal** |
| --- | --- | --- | --- | --- | --- | --- | --- |
| cluster 1286c | 247 | 522 | 2.12 | 100 | 0.00021 | 0.37617 | 0.45056 |
| cluster 1202 | 57770 | 42369 | -1.36 | 75.97 | 0.00055 | 0.37617 | 1 |
| cluster 2512 | 3603 | 2303 | -1.56 | 75.97 | 0.00081 | 0.37617 | 1 |
| cluster 956b | 2129 | 3173 | 1.49 | 100 | 0.00092 | 0.37617 | 1 |
| cluster 1901a | 475 | 272 | -1.75 | 75.61 | 0.00097 | 0.37617 | 1 |
| cluster 1901b | 1907 | 1265 | -1.51 | 75.61 | 0.00103 | 0.37617 | 1 |
| cluster 677 | 1951 | 1368 | -1.43 | 75.97 | 0.00124 | 0.38836 | 1 |
| cluster 3003 | 15919 | 11107 | -1.43 | 75.97 | 0.00190 | 0.52214 | 1 |
| cluster 502 | 3700 | 2679 | -1.38 | 75.61 | 0.00224 | 0.54724 | 1 |
| cluster 481 | 61460 | 49041 | -1.25 | 75.97 | 0.00264 | 0.57865 | 1 |
| cluster 2416 | 1020 | 1447 | 1.42 | 100 | 0.00407 | 0.78545 | 1 |
| cluster 1163 | 9610 | 7140 | -1.35 | 75.97 | 0.00464 | 0.78545 | 1 |
| cluster 3028 | 7698 | 5965 | -1.29 | 75.97 | 0.00473 | 0.78545 | 1 |
| cluster 301g | 231 | 112 | -2.07 | 75.61 | 0.00532 | 0.78545 | 1 |
| cluster 2767a | 24 | 53 | 2.22 | 100 | 0.00537 | 0.78545 | 1 |
| cluster 705a | 592 | 411 | -1.44 | 75.61 | 0.00624 | 0.85551 | 1 |
| cluster 1064 | 2939 | 2285 | -1.29 | 75.97 | 0.00696 | 0.86278 | 1 |
| cluster 597c | 3553 | 4739 | 1.33 | 100 | 0.00724 | 0.86278 | 1 |
| cluster 2443ff | 909 | 631 | -1.44 | 75.97 | 0.00792 | 0.86278 | 1 |
| cluster 170d | 65 | 31 | -2.10 | 75.61 | 0.00825 | 0.86278 | 1 |
| cluster 680a | 64 | 29 | -2.16 | 75.61 | 0.00918 | 0.86278 | 1 |
| cluster 2960 | 3610 | 4777 | 1.32 | 100 | 0.00919 | 0.86278 | 1 |
| cluster 2846 | 3049 | 2341 | -1.30 | 75.97 | 0.00935 | 0.86278 | 1 |
| cluster 1197 | 3294 | 2472 | -1.33 | 75.97 | 0.00985 | 0.86278 | 1 |
| cluster 956c | 891 | 1315 | 1.48 | 100 | 0.01082 | 0.86278 | 1 |
| cluster 230 | 7391 | 5747 | -1.29 | 75.97 | 0.01157 | 0.86278 | 1 |
| cluster 173 | 18 | 40 | 2.25 | 100 | 0.01212 | 0.86278 | 1 |
| cluster 2598 | 6665 | 8485 | 1.27 | 100 | 0.01260 | 0.86278 | 1 |
| cluster 2870a | 1177 | 903 | -1.30 | 75.97 | 0.01261 | 0.86278 | 1 |
| cluster 1515 | 5915 | 4556 | -1.30 | 75.97 | 0.01270 | 0.86278 | 1 |
| cluster 1731 | 2442 | 3244 | 1.33 | 100 | 0.01334 | 0.86278 | 1 |
| cluster 2482b | 6785 | 8697 | 1.28 | 100 | 0.01370 | 0.86278 | 1 |
| cluster 3030b | 582 | 431 | -1.35 | 75.97 | 0.01473 | 0.86278 | 1 |
| cluster 348 | 55363 | 43066 | -1.29 | 75.97 | 0.01477 | 0.86278 | 1 |
| cluster 109 | 19599 | 14875 | -1.32 | 75.97 | 0.01507 | 0.86278 | 1 |
| cluster 154 | 1247 | 955 | -1.31 | 75.97 | 0.01554 | 0.86278 | 1 |
| cluster 356 | 1213 | 950 | -1.28 | 75.97 | 0.01555 | 0.86278 | 1 |
| cluster 148 | 59239 | 49514 | -1.20 | 75.97 | 0.01591 | 0.86278 | 1 |
| cluster 1055 | 1725 | 2380 | 1.38 | 100 | 0.01652 | 0.86278 | 1 |
| cluster 956d | 3055 | 3988 | 1.31 | 100 | 0.01658 | 0.86278 | 1 |
| cluster 700 | 61093 | 51683 | -1.18 | 75.97 | 0.01667 | 0.86278 | 1 |
| cluster 2228 | 583 | 806 | 1.38 | 100 | 0.01723 | 0.86278 | 1 |
| cluster 2971 | 1819 | 2292 | 1.26 | 100 | 0.01742 | 0.86278 | 1 |
| cluster 1559 | 1253 | 987 | -1.27 | 75.97 | 0.01774 | 0.86278 | 1 |
| cluster 1690 | 1627 | 2075 | 1.28 | 100 | 0.01795 | 0.86278 | 1 |
| cluster 2508 | 1800 | 2279 | 1.27 | 100 | 0.01827 | 0.86278 | 1 |
| cluster 495 | 1435 | 1806 | 1.26 | 100 | 0.01883 | 0.86278 | 1 |
| cluster 730 | 443 | 634 | 1.43 | 100 | 0.01901 | 0.86278 | 1 |
| cluster 2118 | 5031 | 3963 | -1.27 | 75.97 | 0.01927 | 0.86278 | 1 |
| cluster 145 | 12820 | 9982 | -1.28 | 75.97 | 0.02000 | 0.87494 | 1 |
| cluster 2029 | 7935 | 9861 | 1.24 | 100 | 0.02094 | 0.87494 | 1 |
| cluster 1451 | 11824 | 15334 | 1.30 | 100 | 0.02124 | 0.87494 | 1 |
| cluster 2777 | 6853 | 8697 | 1.27 | 100 | 0.02162 | 0.87494 | 1 |
| cluster 597a | 205 | 314 | 1.53 | 100 | 0.02319 | 0.87494 | 1 |
| cluster 2976 | 6458 | 5210 | -1.24 | 75.97 | 0.02365 | 0.87494 | 1 |
| cluster 2033 | 11679 | 9363 | -1.25 | 75.97 | 0.02420 | 0.87494 | 1 |
| cluster 58 | 5524 | 4299 | -1.29 | 75.97 | 0.02421 | 0.87494 | 1 |
| cluster 616d | 1243 | 967 | -1.29 | 75.97 | 0.02491 | 0.87494 | 1 |
| cluster 2599b | 45 | 78 | 1.74 | 100 | 0.02520 | 0.87494 | 1 |
| cluster 1389a | 1941 | 1516 | -1.28 | 75.97 | 0.02567 | 0.87494 | 1 |
| cluster 1152 | 4412 | 3450 | -1.28 | 75.97 | 0.02570 | 0.87494 | 1 |
| cluster 577 | 1189 | 1541 | 1.30 | 100 | 0.02592 | 0.87494 | 1 |
| cluster 2746c | 420 | 286 | -1.47 | 75.97 | 0.02605 | 0.87494 | 1 |
| cluster 62b | 2882 | 3658 | 1.27 | 100 | 0.02653 | 0.87494 | 1 |
| cluster 450 | 5272 | 6467 | 1.23 | 100 | 0.02731 | 0.87494 | 1 |
| cluster 1913q | 1969 | 2466 | 1.25 | 100 | 0.02753 | 0.87494 | 1 |
| cluster 2201e | 39 | 91 | 2.33 | 100 | 0.02762 | 0.87494 | 1 |
| cluster 2635b | 393 | 268 | -1.47 | 75.97 | 0.02769 | 0.87494 | 1 |
| cluster 2222a | 1352 | 1673 | 1.24 | 100 | 0.02802 | 0.87494 | 1 |
| cluster 814b | 955 | 1267 | 1.33 | 100 | 0.02808 | 0.87494 | 1 |
| cluster 814a | 1010 | 1371 | 1.36 | 100 | 0.02962 | 0.87494 | 1 |
| cluster 161 | 842 | 1068 | 1.27 | 100 | 0.02996 | 0.87494 | 1 |
| cluster 915a | 6491 | 7919 | 1.22 | 100 | 0.03053 | 0.87494 | 1 |
| cluster 2745b | 2137 | 2684 | 1.26 | 100 | 0.03070 | 0.87494 | 1 |
| cluster 2910c | 34453 | 41343 | 1.20 | 100 | 0.03171 | 0.87494 | 1 |
| cluster 2845 | 2819 | 2271 | -1.24 | 75.97 | 0.03179 | 0.87494 | 1 |
| cluster 2761 | 14855 | 11714 | -1.27 | 75.97 | 0.03188 | 0.87494 | 1 |
| cluster 1485 | 207 | 308 | 1.49 | 100 | 0.03202 | 0.87494 | 1 |
| cluster 1864 | 7091 | 8578 | 1.21 | 100 | 0.03249 | 0.87494 | 1 |
| cluster 3006 | 2658 | 3274 | 1.23 | 100 | 0.03363 | 0.87494 | 1 |
| cluster 171c | 74 | 38 | -1.95 | 75.97 | 0.03389 | 0.87494 | 1 |
| cluster 248 | 7793 | 9605 | 1.23 | 100 | 0.03405 | 0.87494 | 1 |
| cluster 2609 | 1194 | 970 | -1.23 | 75.97 | 0.03420 | 0.87494 | 1 |
| cluster 2729c | 1140 | 1488 | 1.31 | 100 | 0.03455 | 0.87494 | 1 |
| cluster 605 | 1610 | 1997 | 1.24 | 100 | 0.03493 | 0.87494 | 1 |
| cluster 2926 | 5467 | 2932 | -1.86 | 75.97 | 0.03544 | 0.87494 | 1 |
| cluster 2980 | 1876 | 1517 | -1.24 | 75.97 | 0.03595 | 0.87494 | 1 |
| cluster 814d | 1137 | 1419 | 1.25 | 100 | 0.03606 | 0.87494 | 1 |
| cluster 1810f | 1566 | 1923 | 1.23 | 100 | 0.03611 | 0.87494 | 1 |
| cluster 2027 | 1696 | 1363 | -1.24 | 75.97 | 0.03643 | 0.87494 | 1 |
| cluster 2536 | 30950 | 25647 | -1.21 | 75.97 | 0.03657 | 0.87494 | 1 |
| cluster 554 | 2825 | 3500 | 1.24 | 100 | 0.03671 | 0.87494 | 1 |
| cluster 2806i | 9198 | 11299 | 1.23 | 100 | 0.03719 | 0.87494 | 1 |
| cluster 1514 | 4071 | 5184 | 1.27 | 100 | 0.03782 | 0.87494 | 1 |
| cluster 2316 | 826 | 1031 | 1.25 | 100 | 0.03846 | 0.87494 | 1 |
| cluster 2769c | 121 | 60 | -2.01 | 75.97 | 0.03909 | 0.87494 | 1 |
| cluster 2985 | 5513 | 4256 | -1.30 | 75.97 | 0.03963 | 0.87494 | 1 |
| cluster 3062b | 457 | 328 | -1.39 | 75.97 | 0.04002 | 0.87494 | 1 |
| cluster 189 | 2789 | 3380 | 1.21 | 100 | 0.04053 | 0.87494 | 1 |
| cluster 1513 | 6991 | 5644 | -1.24 | 75.97 | 0.04067 | 0.87494 | 1 |
| cluster 1913c | 839 | 654 | -1.28 | 75.97 | 0.04118 | 0.87494 | 1 |
| cluster 2443y | 1479 | 1829 | 1.24 | 100 | 0.04162 | 0.87494 | 1 |
| cluster 2111 | 14271 | 17483 | 1.23 | 100 | 0.04201 | 0.87494 | 1 |
| cluster 301b | 144 | 91 | -1.58 | 75.97 | 0.04234 | 0.87494 | 1 |
| cluster 286 | 1158 | 1435 | 1.24 | 100 | 0.04273 | 0.87494 | 1 |
| cluster 2059e | 2667 | 3335 | 1.25 | 100 | 0.04296 | 0.87494 | 1 |
| cluster 1778 | 3785 | 2962 | -1.28 | 75.97 | 0.04391 | 0.87494 | 1 |
| cluster 843b | 236 | 337 | 1.43 | 100 | 0.04465 | 0.87494 | 1 |
| cluster 445 | 31583 | 25683 | -1.23 | 75.97 | 0.04513 | 0.87494 | 1 |
| cluster 337 | 1086 | 1344 | 1.24 | 100 | 0.04537 | 0.87494 | 1 |
| cluster 3002 | 11809 | 9659 | -1.22 | 75.97 | 0.04540 | 0.87494 | 1 |
| cluster 636f | 261 | 351 | 1.35 | 100 | 0.04556 | 0.87494 | 1 |
| cluster 2858 | 935 | 724 | -1.29 | 75.97 | 0.04583 | 0.87494 | 1 |
| cluster 1407 | 3094 | 2555 | -1.21 | 75.97 | 0.04600 | 0.87494 | 1 |
| cluster 2332 | 19229 | 15662 | -1.23 | 75.97 | 0.04624 | 0.87494 | 1 |
| cluster 1849 | 1766 | 2201 | 1.25 | 100 | 0.04647 | 0.87494 | 1 |
| cluster 2968 | 6263 | 5144 | -1.22 | 75.97 | 0.04666 | 0.87494 | 1 |
| cluster 396a | 24802 | 29649 | 1.20 | 100 | 0.04711 | 0.87591 | 1 |
| cluster 2613 | 1262 | 1023 | -1.23 | 75.97 | 0.04788 | 0.88277 | 1 |
| cluster 2163 | 847 | 629 | -1.35 | 75.97 | 0.04970 | 0.90866 | 1 |
