# Supplementary material for: Development and Validation of an Haemophilus influenzae Supragenome Hybridization (SGH) Array for Transcriptomic Analyses
Source: PLoS One. 2014 Oct 7;9(10):e105493. doi: 10.1371/journal.pone.0105493 (PMC4188559; doi:10.1371/journal.pone.0105493)
Supplement: Table S3 — False positives using a fold change cutoff of >1.5. Condition 4, replicate B, chip 1 and chip 2 were compared. Raw expression values are shown. FDR: False discovery rate, BH: Benjamini-Hochberg, Bon. pVal: Bonferroni-corrected p-value. (DOCX) [file pone.0105493.s033.docx]

Table S3. False positives using a fold change cutoff of >1.5

Condition 4, replicate B, chip 1 and chip 2 were compared. Raw expression values are shown. FDR: False discovery rate, BH: Benjamini-Hochberg, Bon. pVal: Bonferroni-corrected p-value

| **Subcluster** | **Chip1 expression** | **Chip2 expression** | **FOLD** | **SAM FDR (%)** | **pVal** | **BH FDR** | **Bon. pVal** |
| --- | --- | --- | --- | --- | --- | --- | --- |
| cluster 680a | 64 | 29 | -2.16 | 75.61 | 0.00918 | 0.86278 | 1 |
| cluster 170d | 65 | 31 | -2.10 | 75.61 | 0.00825 | 0.86278 | 1 |
| cluster 896 | 125 | 60 | -2.08 | 75.97 | 0.07198 | 0.99155 | 1 |
| cluster 301g | 231 | 112 | -2.07 | 75.61 | 0.00532 | 0.78545 | 1 |
| cluster 2769c | 121 | 60 | -2.01 | 75.97 | 0.03909 | 0.87494 | 1 |
| cluster 171c | 74 | 38 | -1.95 | 75.97 | 0.03389 | 0.87494 | 1 |
| cluster 2926 | 5467 | 2932 | -1.86 | 75.97 | 0.03544 | 0.87494 | 1 |
| cluster 206d | 172 | 98 | -1.75 | 75.97 | 0.05233 | 0.92314 | 1 |
| cluster 1901a | 475 | 272 | -1.75 | 75.61 | 0.00097 | 0.37617 | 1 |
| cluster 1031 | 150 | 92 | -1.63 | 75.97 | 0.06302 | 0.99155 | 1 |
| cluster 301b | 144 | 91 | -1.58 | 75.97 | 0.04234 | 0.87494 | 1 |
| cluster 2512 | 3603 | 2303 | -1.56 | 75.97 | 0.00081 | 0.37617 | 1 |
| cluster 3078 | 59 | 38 | -1.55 | 75.97 | 0.10672 | 0.99155 | 1 |
| cluster 1279a | 392 | 254 | -1.54 | 94.77 | 0.09571 | 0.99155 | 1 |
| cluster 680b | 44 | 29 | -1.52 | 75.61 | 0.05242 | 0.92314 | 1 |
| cluster 301c | 135 | 89 | -1.52 | 75.61 | 0.06579 | 0.99155 | 1 |
| cluster 746 | 63 | 41 | -1.52 | 75.97 | 0.09825 | 0.99155 | 1 |
| cluster 170a | 39 | 26 | -1.51 | 75.97 | 0.13392 | 0.99155 | 1 |
| cluster 2788i | 109 | 72 | -1.51 | 75.61 | 0.10093 | 0.99155 | 1 |
| cluster 1901b | 1907 | 1265 | -1.51 | 75.61 | 0.00103 | 0.37617 | 1 |
| cluster 734b | 82 | 123 | 1.50 | 100 | 0.07970 | 0.99155 | 1 |
| cluster 597a | 205 | 314 | 1.53 | 100 | 0.02319 | 0.87494 | 1 |
| cluster 339b | 44 | 69 | 1.56 | 100 | 0.09244 | 0.99155 | 1 |
| cluster 2808c | 66 | 102 | 1.56 | 100 | 0.14242 | 0.99155 | 1 |
| cluster 2042a | 51 | 81 | 1.58 | 100 | 0.09669 | 0.99155 | 1 |
| cluster 2599b | 45 | 78 | 1.74 | 100 | 0.02520 | 0.87494 | 1 |
| cluster 1286c | 247 | 522 | 2.12 | 100 | 0.00021 | 0.37617 | 0.45056 |
| cluster 2767a | 24 | 53 | 2.22 | 100 | 0.00537 | 0.78545 | 1 |
| cluster 173 | 18 | 40 | 2.25 | 100 | 0.01212 | 0.86278 | 1 |
| cluster 2201e | 39 | 91 | 2.33 | 100 | 0.02762 | 0.87494 | 1 |
